# Proteomic insights into extracellular vesicles in ALS for therapeutic potential of Ropinirole and biomarker discovery

**DOI:** 10.1186/s41232-024-00346-1

**Published:** 2024-07-12

**Authors:** Chris Kato, Koji Ueda, Satoru Morimoto, Shinichi Takahashi, Shiho Nakamura, Fumiko Ozawa, Daisuke Ito, Yugaku Daté, Kensuke Okada, Naoki Kobayashi, Jin Nakahara, Hideyuki Okano

**Affiliations:** 1 Keio University Regenerative Medicine Research Center, Kanagawa, 210-0821 Japan; 2 Department of Physiology, Keio University School of Medicine, Tokyo, 160-8582 Japan; 3 Division of Neurodegenerative Disease Research, Tokyo Metropolitan Institute for Geriatrics and Gerontology, Tokyo, Japan; 4 Cancer Proteomics Group, Cancer Precision Medicine Center, Japanese Foundation for Cancer Research, Tokyo, 135-8550 Japan; 5 Department of Neurology and Cerebrovascular Medicine, Saitama Medical University International Medical Center, Saitama, 350-1298 Japan; 6 Department of Neurology, Keio University School of Medicine, Tokyo, 160-8582 Japan

**Keywords:** Amyotrophic lateral sclerosis (ALS), Extracellular vesicle, Cerebrospinal fluid (CSF), Blood, Proteomics, Time-series, Induced pluripotent stem cells (iPSCs), Motor neurons, Astrocytes

## Abstract

**Background:**

Extracellular vesicles (EVs) hold the potential for elucidating the pathogenesis of amyotrophic lateral sclerosis (ALS) and serve as biomarkers. Notably, the comparative and longitudinal alterations in the protein profiles of EVs in serum (sEVs) and cerebrospinal fluid (CSF; cEVs) of sporadic ALS (SALS) patients remain uncharted. Ropinirole hydrochloride (ROPI; dopamine D2 receptor [D2R] agonist), a new anti-ALS drug candidate identified through induced pluripotent stem cell (iPSC)-based drug discovery, has been suggested to inhibit ALS disease progression in the Ropinirole Hydrochloride Remedy for Amyotrophic Lateral Sclerosis (ROPALS) trial, but its mechanism of action is not well understood. Therefore, we tried to reveal longitudinal changes with disease progression and the effects of ROPI on protein profiles of EVs.

**Methods:**

We collected serum and CSF at fixed intervals from ten controls and from 20 SALS patients participating in the ROPALS trial. Comprehensive proteomic analysis of EVs, extracted from these samples, was conducted using liquid chromatography/mass spectrometer (LC/MS). Furthermore, we generated iPSC-derived astrocytes (iPasts) and performed RNA sequencing on astrocytes with or without ROPI treatment.

**Results:**

The findings revealed notable disparities yet high congruity in sEVs and cEVs protein profiles concerning disease status, time and ROPI administration. In SALS, both sEVs and cEVs presented elevated levels of inflammation-related proteins but reduced levels associated with unfolded protein response (UPR). These results mirrored the longitudinal changes after disease onset and correlated with the revised ALS Functional Rating Scale (ALSFRS-R) at sampling time, suggesting a link to the onset and progression of SALS. ROPI appeared to counteract these changes, attenuating inflammation-related protein levels and boosting those tied to UPR in SALS, proposing an anti-ALS impact on EV protein profiles. Reverse translational research using iPasts indicated that these changes may partly reflect the DRD2-dependent neuroinflammatory inhibitory effects of ROPI. We have also identified biomarkers that predict diagnosis and disease progression by machine learning-driven biomarker search.

**Conclusions:**

Despite the limited sample size, this study pioneers in reporting time-series proteomic alterations in serum and CSF EVs from SALS patients, offering comprehensive insights into SALS pathogenesis, ROPI-induced changes, and potential prognostic and diagnostic biomarkers.

**Supplementary Information:**

The online version contains supplementary material available at 10.1186/s41232-024-00346-1.

## Background

Amyotrophic lateral sclerosis (ALS), a fatal neurodegenerative disease, is characterized by the degeneration and subsequent loss of function of both upper and lower motor neurons. The disease progresses rapidly, with median survival from onset to death ranging from 20 to 48 months [1]. Approximately 90% of ALS patients present with a sporadic onset (sporadic ALS; SALS), underscoring the highly variable nature of its pathogenesis [2].

Extracellular vesicles (EVs), which are ubiquitously secreted by most cell types, have been identified in numerous body fluids such as blood and cerebrospinal fluid (CSF) [3]. EVs encapsulate biomolecules such as proteins, nucleic acids, and lipids, serving as conduits for cellular communication [3, 4]. Given their lipid bilayer composition and stability in the circulation, the potential contribution of EVs to the pathogenesis of diseases such as cancer and neurodegenerative diseases, and their potential as biomarkers, has generated considerable interest [3–6]. Numerous studies have attempted to identify ALS biomarkers by studying EVs [5, 7]. The importance of longitudinal analysis for understanding this disease cannot be overemphasized [8], but longitudinal analysis of EV protein composition in ALS is markedly understudied [9]. Ropinirole hydrochloride (ROPI), a dopamine D2 receptor agonist, is a widely used drug approved more than 15 years ago for Parkinson’s disease treatment [10]. Subsequently, ROPI was identified as a novel drug candidate for ALS treatment through drug screening using induced pluripotent stem cell-derived spinal motor neurons (iPSC-MNs) from ALS patients [11–14]. We conducted the Ropinirole Hydrochloride Remedy for Amyotrophic Lateral Sclerosis (ROPALS) trial, a single-center, randomized, double-blind, placebo-controlled trial, which suggested that ROPI may inhibit disease progression in SALS patients [15, 16].

In the present study, we collected serum and CSF samples from 10 controls and 20 SALS patients enrolled in the ROPALS trial at predefined time points. We thoroughly and quantitatively examined the protein profiles of EVs in the serum (sEVs) and CSF (cEVs) of controls and SALS patients to identify longitudinal characteristic changes in SALS pathogenesis, changes induced by ROPI administration, and potential biomarkers for pathological indicators and diagnosis. Our study revealed that sEVs and cEVs from SALS patients exhibit increases in inflammation-related proteins, especially the complement and coagulation cascades, and decreases in proteins of the unfolded protein response (UPR), diverging from controls, and aligning with disease progression over time.

## Methods

### Study participants

This study included 20 SALS patients enrolled in the ROPALS trial conducted at Keio University Hospital, Japan. The inclusion and exclusion criteria and other details are included in the ROPALS trial protocol [15]. Additionally, serum and CSF samples collected from 10 non-neurodegenerative Japanese adults (controls) were obtained from the NCNP Biobank, a member of the National Center Biobank Network [17].

### ROPALS trial and blood/CSF sampling

**Fig. 1:**
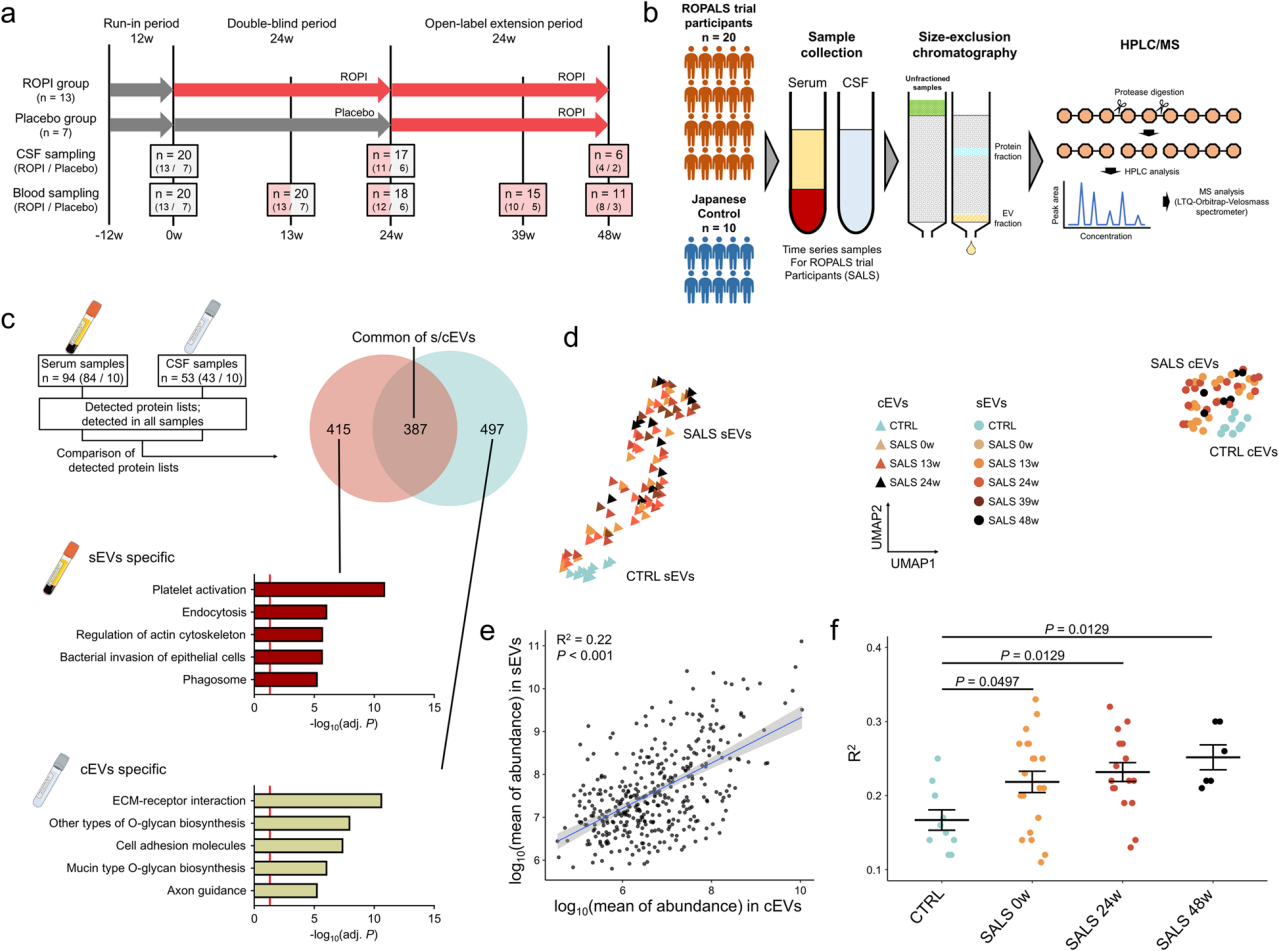
Protein profiles of sEVs and cEVs differ between controls and SALS patients. **a** Overview of the ROPALS trial and timing of serum and CSF sampling. **b** Scheme of the study: EVs were extracted from serum and CSF collected from controls (*n* = 10, point-in-time collection, from the NCNP Biobank) and SALS patients (*n* = 20, longitudinal collection) participating in the ROPALS trial and subjected to comprehensive quantitative proteomic analysis using Orbitrap Fusion Lumos mass spectrometer. **c** Comparison of protein profiles in sEVs and cEVs derived from controls or SALS patients. The Venn diagram shows a comparison of proteins within sEVs and cEVs detected in all samples (using samples from both controls and SALS patients). Bar graphs show the KEGG pathway analysis results for proteins specifically identified in sEVs and cEVs. **d** UMAP analysis result for all EVs, sEVs and cEVs. **e** Correlation plots and Pearson’s correlation analysis results for proteins detected in both sEVs and cEVs with respect to their average level in sEVs and cEVs. Each point represents a single protein. **f** Correlation analysis results of protein profiles detected in both sEVs and cEVs collected from the same subject at the same time. Each point represents the correlation coefficient comparing the content of each protein in sEVs and cEVs collected from the same subject at the same time (Dunnett's test, controls-0w: adj. *P* = 0.0497, controls-24w: adj. *P* = 0.0129, controls-48w: adj. *P* = 0.0129, mean ± s.d.)

In the ROPALS trial, 20 SALS patients without any previously reported mutations of ALS underwent 60 weeks of follow-up consisting of a 12-week run-in period, 24-week double-blind period, and 24-week open-label extension period. SALS patients were assigned to the ROPI treatment (ROPI group) and placebo groups (13 and seven patients, respectively). The 13 patients in the ROPI group received ROPI for the entire 48-week intervention period, while the seven patients in the placebo group received placebo for the first 24 weeks of the 48-week intervention period (double-blind period) and ROPI for the second 24 weeks (open-label extension period) (Fig. 1a).

The first week of ROPI/placebo administration was considered week 0 (0w). Blood samples were collected from patients at 0w (*n* = 20), 13w (*n* = 20), 24w (*n* = 18), 39w (*n* = 15), and 48w (*n* = 11). Similarly, CSF was collected at 0w (*n* = 20), 24w (*n* = 17), and 48w (*n* = 6). The sample collection and preservation methods were in accordance with the clinical trial protocol for SALS patient samples [15] and the NCNP Biobank methods for control samples [17].

### EV isolation and mass spectrometry

EVs released into serum, CSF, and induced iPSC-derived astrocyte (iPast) culture medium were collected using EV Second L70 columns (GL Sciences, Tokyo, Japan) according to the manufacturer’s instructions. Proteins in EVs were reduced with 10 mM TCEP at 100 °C for 10 min, alkylated with 50 mM iodoacetamide at room temperature for 45 min, and digested on beads with Trypsin/Lys-C Mix (Promega) at 37 °C for 12 h. The resulting peptides were analysed with Orbitrap Fusion Lumos mass spectrometer (Thermo Scientific) and UltiMate 3000 RSLC nano-flow HPLC system (Thermo Scientific). Peptides were enriched using a μ-Precolumn (0.3 mm i.d. × 5 mm, 5 μm, Thermo Scientific) and separated on an AURORA column (0.075 mm i.d. × 250 mm, 1.6 μm, Ion Opticks Pty Ltd., Australia) using a two-step gradient: 2–40% acetonitrile for 110 min, followed by 40–95% acetonitrile with 0.1% formic acid for 5 min. The analytical parameters of Orbitrap Fusion Lumos were as follows: full scan resolution, 50,000; Scan range (m/z), 350–1500; full scan maximum injection time, 50 ms; full scan AGC target, 4 × 105; dynamic exclusion duration, 30 s; data-dependent MS/MS acquisition cycle time, 2 s; activation type, HCD; MS/MS detector, ion trap; maximum MS/MS injection time, 35 ms; AGC target of MS/MS, 1 × 104. The MS/MS spectra were searched against the *Homo sapiens* protein sequence database in SwissProt using Proteome Discoverer 2.5 software (Thermo Scientific), and the peptide identification filters were set as “false discovery rate < 1%.” Label-free relative quantification analysis of proteins was performed using the default parameters of Minora Feature Detector node, Feature Mapper node, and Precursor Ions Quantifier node in Proteome Discoverer 2.5 (Fig. 1b).

### Comprehensive quantification of EV proteins

Longitudinal samples from 20 SALS patients were measured in the first batch, and samples from 10 controls and three SALS patients (0w samples, sEV ID; RPR-01–31/32/33, cEV ID; RPR-01–24/25/33) were measured in the second batch. Three identical samples were measured in two batches each of sEVs and cEVs to eliminate possible batch effects of processing samples from different batches. The second batch measurements were corrected using the following equations.

$$\text{Protein A measurements in first batch}={X}_{i} (i=\text{1,2},3)$$

$$\text{Protein A measurements in second batch}={{X}{\prime}}_{i } (i=\text{1,2},3)$$

$$\text{Protein A measurement in second batch}=Y$$

$$\text{Protein A correction value in second batch}=Z$$

$Z=Y/\frac{1}{3}\left(\sum_{i=1}^{3}\frac{{X{\prime}}_{i}}{{X}_{i}}\right)$ For downstream analysis, all protein measurements were log_10_-transformed after batch-to-batch correction so that the data followed a normal distribution.

### Machine learning

All machine learning analyses were performed in Python (version 3.8) using scikit-learn (version 1.2.1) and xgboost (version 1.7.5) [18–20]. Given the limited sample size and presence of unbalanced classes, a learning framework was constructed (Fig. 8a). In the oversampling step, the unbalanced dataset of 13 samples (control samples: *n* = 10, SALS patient samples: *n* = 3) measured in the first batch was expanded to a balanced dataset of 19 samples (control samples: *n* = 10, SALS patient samples: *n* = 9) using the SMOTE algorithm of Imbalanced-learn (version 0.0) [21]. Data from all 19 samples were randomly assigned to a training dataset including 13 samples (68.4%) and a test dataset including six samples (31.6%), considering control and SALS patient samples. Validation was performed using 84 serum and 43 CSF samples measured in the second batch. Of these samples, 33 serum and 13 CSF samples were from ROPI-naive patients, and the remaining samples were from patients who had received ROPI (ROPI-exposed). We used scikit-learn’s GridSearchCV for parameter tuning with twofold cross-validation and RFECV for recursive feature elimination in the feature selection phase [19], with a random seed of 111.

### iPSC-derived astrocyte and spinal motor neuron study

#### iPSC-derived astrocyte study

##### Cell lines and culture

We used iPSCs derived from one healthy control (WD39; CVCL_Y528) and differentiated human iPasts according to our previously reported method [22].

### Ribonucleic acid sequencing (RNA-seq)

The amount and quality of RNA was confirmed using an Agilent TapeStation (Agilent Technologies, CA, USA). RNA was amplified using a SMART-Seq v4 Ultra Low Input RNA Kit for Sequencing (Takara Bio, Shiga, Japan), and a DNA library was prepared using a Nextera XT DNA Library Prep Kit (Illumina, CA, USA). RNA-seq was performed using a NovaSeq system (Illumina). All procedures were performed by Takara Bio.

### iPSC-MN analysis

RNA-seq data from healthy subject-derived iPSC-MNs (t84h) were obtained from deposited data in the NCBI Gene Expression Omnibus (GEO) database, GEO accession number GSE209696 [15].

### Analysis tools

All analyses except for the machine learning portions were performed using R (version 4.1.2) [23]. Effsize (version 0.8.1) [24] was used to calculate effect sizes. Dunnett’s test was performed using the aov() function and multcomp (version 1.4.0) [25]. The prcomp() function was used for PCA, and “scale” was set to TRUE as an argument, as recommended in the official documentation.

For RNA-seq analysis, fastp (version 0.23.2, all arguments set to default) was used for quality control of fastq files [26]. Read counts were quantified using Salmon (version 1.6, all arguments set to default) for samples that passed quality control [27]. The Salmon reference was gencode, release 42 (GRCh38.p13). The Salmon output file was converted to a count file using the R package tximport (version 1.22.0) [28]. DESeq2 (version 1.34.0) from the R package was used for group comparisons [29].

Gene Ontology (GO) (biological pathway, BP; cellular component, CC; molecular function, MF) enrichment and Kyoto Encyclopedia of Genes and Genomes (KEGG) pathway analyses were performed using g:Profiler [30–35]. Adjusted *P* (adj. *P*) values calculated by statistical analysis were entered in decreasing order, and the “Ordered query” was checked. According to Reimond et al. [36], “term size” was set to “5–350.” lsa (version 0.73.3) was used for cosine similarity analysis [37]. The nls() function was used to approximate changes in the revised ALS Functional Rating Scale (ALSFRS-R), a key clinical indicator of ALS symptoms, during the ROPALS study using the following model equation [15]:

$$ALSFRSR=-\text{exp}\left(a*week\right)+b$$

For GO (BP) and KEGG pathway analysis visualization, the output files in the GEM file were clustered and annotated by g:profiler using Cytoscape (version 3.9.1) [38], EnrichmentMap (version 3.3.4) [39], and AutoAnnotate (version 1.4.0) [40].

### Statistics

Demographic data were analysed using Student’s *t* test (age) and the χ2 test (sex and race). Student’s *t* test was used to compare log_10_-transformed protein measurement levels between two groups. The Storey method was used for multiple testing correction. Two-way ANOVA followed by Bonferroni’s multiple-comparisons test was used for longitudinal two-group analysis of the effects of treatments (placebo vs ROPI) and time on response variables (protein abundances) using the ez package [41]. Estimated marginal means were calculated using the emmeans package [42]. Pairwise comparisons were performed using these estimated marginal means, and *P* values were adjusted using Bonferroni's method to control for the family-wise error rate. Pearson’s product-moment correlation coefficient was used for correlation analyses unless otherwise noted. *P* or adj. *P* values were calculated using two-tailed tests, and values less than 0.05 indicated statistical significance. This cut-off was also used to identify significantly differentially abundant proteins (DAPs) and differentially expressed genes (DEGs). As described in the effsize (version 0.8.1) package manual [24], Cohen’s *d* was considered negligible, small, medium, or large when the absolute value was < 0.2, < 0.5, < 0.8, or ≧ 0.8, respectively.

## Results

### Study participants

Ten controls and 20 SALS patients participated in this study. There were no statistically significant differences in sex (*χ2*(1) = 0.1563, *P* = 0.6926) or race (*χ2*(1) = NA, *P* > 0.9999) between the two groups, but SALS patients were significantly older than controls (*t*(27.14) = 3.8354, *P* < 0.001). Demographic and clinical data of the participants are shown in Supplementary Table S1.

### Protein compositions of sEVs and cEVs differed from each other and from controls and SALS patients

The protein compositions of EVs in body fluids were compared between controls and SALS patients. Serum and CSF were collected over time from 20 SALS patients enrolled in the ROPALS trial (Fig. 1a). EV fractions were then extracted and subjected to comprehensive quantitative proteomic analysis (Fig. 1b). Typical EV markers such as CD9 and Alix were detected in all samples [43], indicating extraction of high-purity EVs (Supplementary Table S2). Additionally, as many as 3719 and 2754 proteins from sEVs and cEVs, respectively, were detected in at least one sample.

To investigate differences in sEV and cEV proteins, we compared the protein types detected in all sEV or cEV samples (using samples from both controls and SALS patients). We identified 802 and 884 proteins from sEVs and cEVs, respectively, of which 387 proteins were commonly present (Fig. 1c, Venn diagram). sEV-specific proteins were mainly associated with Platelet activation (KEGG pathway; KP, adj. *P* < 0.001), whereas cEV-specific proteins were mainly associated with ECM-receptor interaction (KP, adj. *P* < 0.001) (Fig. 1c, bar graphs). To determine the compositional differences between sEVs and cEVs, uniform manifold approximation and projection (UMAP) analysis was performed on proteins detected in all samples of both groups. sEVs and cEVs were clearly divided into two groups, suggesting that the sEV and cEV protein compositions markedly differ (Fig. 1d). Furthermore, UMAP analysis of sEVs and cEVs showed that the distributions of both groups differed from those from controls and from SALS patients, but the changes over time were smaller than the differences because of the presence of disease.

Next, we compared the levels of proteins detected in both sEVs and cEVs. The levels of each protein present in both groups were significantly positively correlated (R^2^ = 0.22, adj. *P* < 0.001) (Fig. 1e), but some proteins were unevenly present in either sEVs or cEVs. Additionally, we performed a correlation analysis of the different protein levels of sEVs and cEVs in each SALS patient at each time point. The coefficients of determination between the protein contents of sEVs and cEVs at 0w, 24w, and 36w were significantly higher in SALS patients than in controls (Dunnett’s test, adj. *P* = 0.0497, 0.0129, and 0.0129, respectively), indicating that the sEV and cEV protein composition was more similar in SALS patients (Fig. 1f). Furthermore, the correlation coefficients tended to increase over time in SALS patients, suggesting that the sEV and cEV composition became more similar over time.

### Comparative analysis of protein composition of EVs from controls and SALS patients

First, to identify the distinct characteristics of protein profiles within sEVs and cEVs derived from ROPI-naive SALS patients, we defined sEV- and cEV-derived SALS-specific proteins as those identified in $\ge$ 90% of sEVs and cEVs from SALS patients and $\le$ 10% of those from controls. We identified 365 EV-derived SALS-specific proteins from sEVs and 398 from cEVs (Supplementary Fig. S1).

SALS-specific proteins from sEVs were suggested to be involved in lipase activity (MF, adj. *P* = 0.002) (Supplementary Fig. S1a). Additionally, SALS-specific proteins from cEVs were associated with immunoglobulin complexes (CC, adj. *P* < 0.001) and antigen binding (MF, adj. *P* < 0.001). Furthermore, SALS-specific proteins from cEVs contained C-reactive protein, suggesting that inflammation may be induced in the CSF in SALS patients (Supplementary Fig. S1b, Supplementary Table S2).

**Fig. 2:**
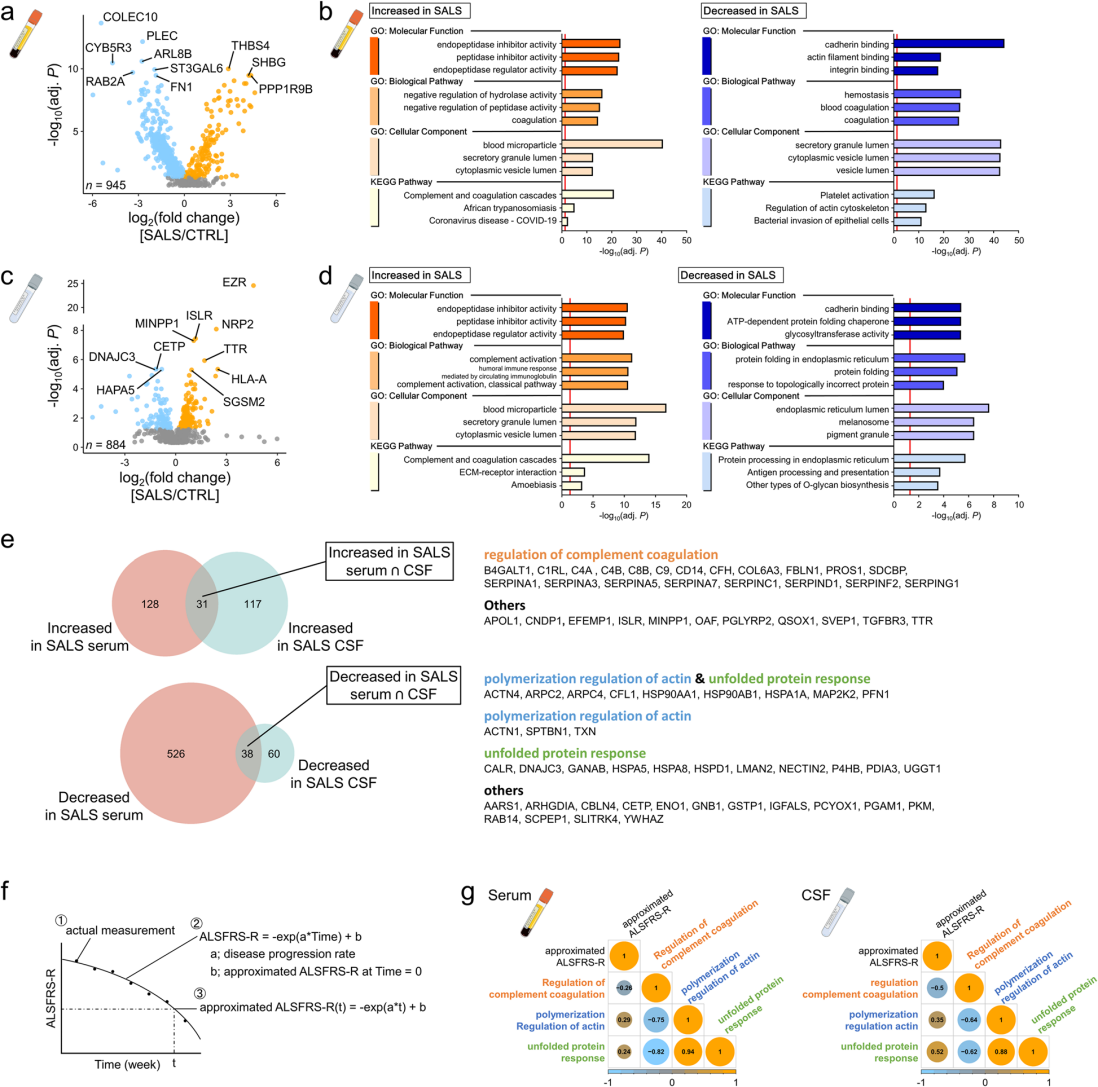
Comparative analysis of protein profiles of EVs in controls and SALS patients. **a**, **c** Volcano plots showing comparisons of proteins within EVs in serum and CSF samples from controls and ROPI-naive SALS patients (sEVs: **a**, cEVs: **c**). Comparisons were made for proteins detected in all samples from controls and ROPI-naive SALS patients. Comparisons between controls and SALS patients were performed using Student’s *t* test on log_10_-transformed data and corrected for the *P* value using Storey's method. **b**, **d** GO (BP, CC, MF) and KEGG pathway analysis results for DAPs (controls vs SALS patients) in sEVs and cEVs (sEVs: **b**, cEVs: **d**). **e** Left: Comparison of DAPs (samples from controls vs samples from SALS patients) in sEVs and cEVs. Right: Lists of proteins commonly decreased/increased in sEVs and cEVs in SALS patients compared with those in controls and their GO (BP) and KEGG pathway clustering analysis results. Letter colors indicate groups (orange: regulation of complement coagulation, blue: polymerization regulation of actin, green: unfolded protein processing, black: other). **f** Schematic representation of disease progression modeling methods and aALSFRS-R calculation. **g** Pearson correlation analysis between the mean levels of each protein belonging to the regulation of complement coagulation, polymerization regulation of actin, and unfolded protein processing groups and the aALSFRS-R at the time of sampling. The size of the circles represents the correlation coefficient

Next, we compared the composition of proteins in sEVs and cEVs from controls and ROPI-naive SALS patients. To improve reproducibility, proteins used for comparative analyses included those detected in all samples from controls and ROPI-naive SALS patients. We identified 723 significantly DAPs in sEVs (Fig. 2a) and 246 in cEVs (Fig. 2c).

DAPs elevated in sEVs (*n* = 159) were associated with the Complement and coagulation cascades (KP, adj. *P* < 0.001), whereas decreased DAPs (*n* = 564) were associated with Platelet activation (KP, adj. *P* < 0.001) (Fig. 2b, Supplementary Fig. S2a). Furthermore, DAPs elevated in cEVs (*n* = 148) and sEVs were associated with the complement and coagulation cascades (KP, adj. *P* < 0.001), and decreased DAPs (*n* = 98) were significantly associated with protein processing in the endoplasmic reticulum (KP, adj. *P* < 0.001) (Fig. 2d, Supplementary Fig. S2b).

Finally, we examined TDP-43, which forms aggregates in motor neurons of ALS patients [44]. Previous studies showed that TDP-43 in plasma, CSF, and cultured cell-derived EVs of SALS patients is elevated compared with that in controls or over time [45–47]. TDP-43 was detected in 0% (0/10) of control and 10.7% (9/84) of SALS patient sEVs and 0% (0/10) of control and 18.6% (8/43) of SALS patient cEVs (Supplementary Table S3). No detection rates were significantly different between controls and SALS patients (sEVs; *χ2*(1) = 0.9800, *P* = 0.3222, cEVs; *χ2*(1) = 0.2705, *P* = 0.6030). Furthermore, TDP-43-positive samples were not consistent over time in the same SALS patients or in serum and CSF (Supplementary Table S3).

### Comparison of DAPs (controls vs. SALS patients) in sEVs and cEVs

Next, we compared DAPs in sEVs and cEVs. Thirty-one DAPs were identified as elevated and 38 as decreased in both sEVs and cEVs in SALS patients (Fig. 2e, left). Functional analysis of each of these DAPs revealed that the DAPs elevated in SALS patients were involved in the regulation of complement coagulation, whereas those that were decreased in SALS patients were involved in regulation of actin polymerization and unfolded protein processing (Fig. 2e, right, the cluster name assigned by AutoAnnotate [40] was “unfolded protein processing,” but it is referenced as the more general term “unfolded protein response (UPR)” hereafter.).

Next, the approximated ALSFRS-R (aALSFRS-R) at the time of sampling was calculated to determine the relationship between the average EV protein content in each category (regulation complement coagulation, polymerization regulation actin, and UPR) and the ALSFRS-R values at the time of sampling. The aALSFRS-R value was obtained by approximating the pathological progression of each patient using $ALSFRS$*-*$R=-\text{exp}\left(a*week\right)+b$ and substituting the number of weeks at the time of sampling (Fig. 2f).

The correlation analysis between the average EV content of each protein category and the aALSFRS-R at the time of sampling showed that the regulation of complement coagulation-related and UPR-related protein group contents were significantly inversely correlated in sEVs (R =  − 0. 82, *P* < 0.001) and cEVs (R =  −0.62, *P* < 0.001) (Fig. 2g). In contrast, the UPR-related protein level in cEVs was significantly correlated with the aALSFRS-R value (R = 0.52, *P* < 0.001), indicating that the UPR-related protein level in cEVs decreased with disease progression.

These results suggest that disease progression in SALS is characterized by an increase in the regulation of complement coagulation-related protein group and a decrease in the UPR-related protein group in EVs.

### ROPI decreases complement and coagulation-related and increases UPR-related proteins in EVs

The log_2_ (fold change) in sEVs and cEVs from 0 to 24w was calculated for each of the 20 SALS patients in the ROPALS trial (13 and 7 in the ROPI and placebo groups, respectively) and compared between the ROPI and placebo groups. We excluded three patients who did not have 24w CSF samples and two patients without 24w serum samples (Fig. 1a). After applying Student’s *t* test and correction for the *P* value using the Storey method, no statistically significant differences were found for any proteins, mainly because the sample number was small relative to the data variability. Therefore, according to the effsize manual [24], a R package for calculating the effect size, the effect size cut-off for Student’s *t* test (Cohen’s *d*) was set to ± 0.5, and proteins for which the effect of ROPI was moderate or greater were defined as effect size-based differentially abundant proteins (es-DAPs).

**Fig. 3:**
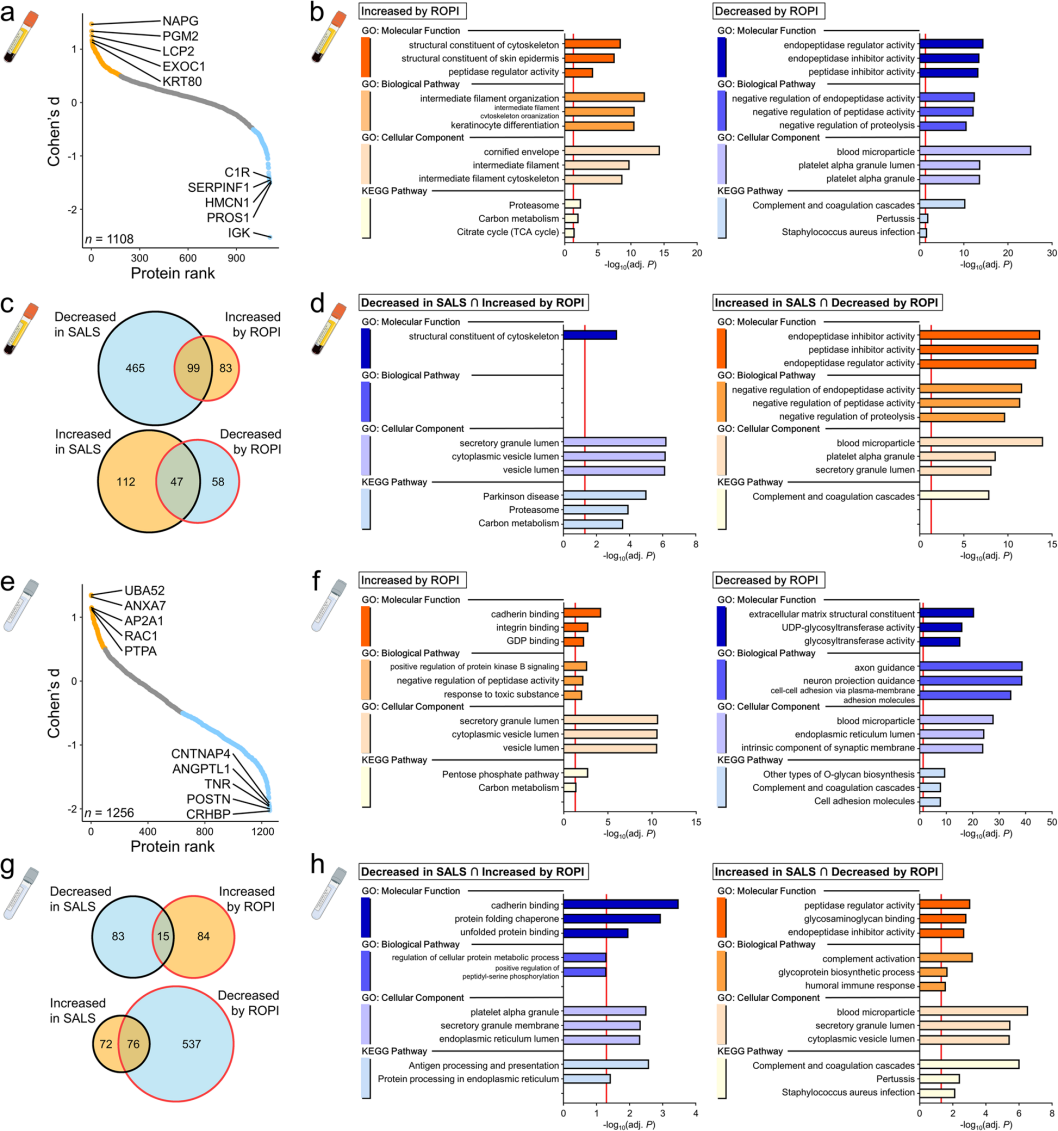
Changes in protein profiles of EVs by ROPI administration. **a**, **e** Rank plot showing Cohen's d comparing the log_2_(fold change) in the ROPI group and placebo group for each patient and each protein in sEVs or cEVs at week 0 and 24 (sEVs: A, cEVs: E). es-DAPs were identified by Cohen’s d using Student’s *t* test with a cut-off value of ± 0.5. **b**, **f** GO (BP, CC, MF) and KEGG pathway analysis results (sEVs: **b**, cEVs: **f**) for es-DAPs (placebo 0–24w vs ROPI 0–24w). **c**, **g** Venn diagram comparing es-DAPs (Placebo 0–24w vs ROPI 0–24w) and DAPs (control samples vs SALS patient samples) in sEVs and cEVs (sEVs: **c**, cEVs: **g**). **d**, **h** GO (BP, CC, MF) and KEGG pathway analysis results (sEVs: **d**, cEVs: **h**) for the protein groups that were increased/decreased in SALS patient samples and with ROPI treatment

Comparisons of sEV and cEV protein content changes from 0–24w in the ROPI and placebo groups identified 287 and 712 es-DAPs, respectively (Fig. 3a, e). In sEVs, es-DAPs (*n* = 182) increased by ROPI treatment were related to Proteasomes (KP, adj. *P* = 0.0034), while es-DAPs (*n* = 105) decreased by ROPI administration were involved in the complement and coagulation cascades (KP, adj. *P* < 0.001) (Fig. 3b). In cEVs, es-DAPs (*n* = 99) increased by ROPI treatment were associated with the Pentose phosphate pathway (KP, adj. *P* = 0.0017), and es-DAPs (*n* = 613) decreased by ROPI were associated with the complement and coagulation cascades (KP, adj. *P* < 0.001) (Fig. 3f).

We further compared DAPs (controls vs. SALS patients) and es-DAPs with and without ROPI treatment (ROPI 0–24w vs placebo 0–24w) (Fig. 3c, g). Proteins with decreased content in SALS patients that were increased by ROPI (sEVs: *n* = 99, cEVs: *n* = 15) were associated with Parkinson’s disease (KP, adj. *P* < 0.001) and Proteasomes (KP, adj. *P* < 0.001) in sEVs (Fig. 3d) and with protein processing in the endoplasmic reticulum (KP, adj. *P* = 0.0366) in cEVs (Fig. 3h). Conversely, proteins with increased content in SALS patients that were decreased by ROPI (sEVs: *n* = 47, cEVs: *n* = 76) were related to the complement and coagulation cascades in both sEVs and cEVs (KP, adj. *P* < 0.001 and < 0.001, respectively) (Fig. 3d, h).

### Protein changes in sEVs and cEVs over time

**Fig. 4:**
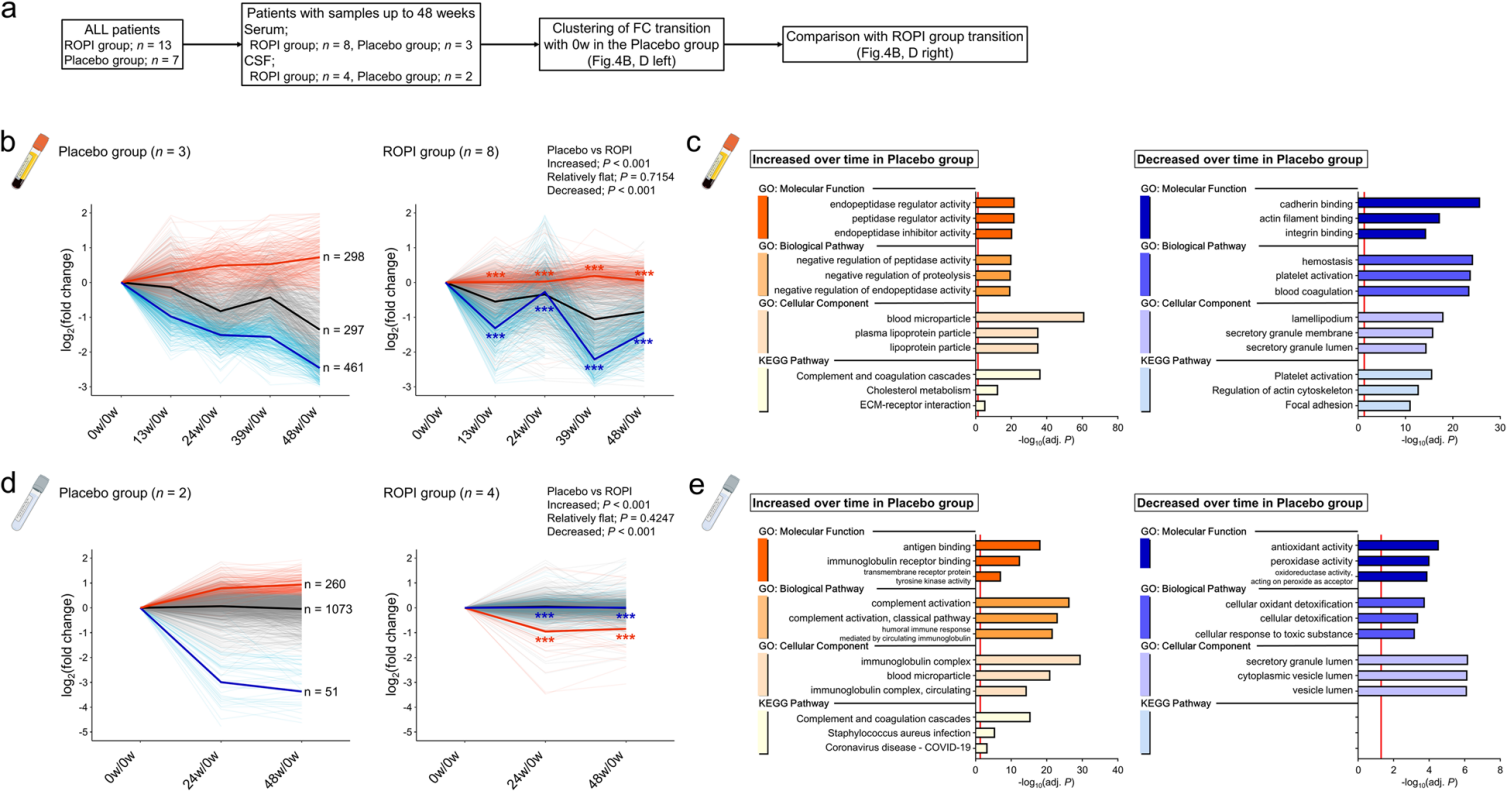
Changes in proteins within EVs over time in SALS patients. **a** Scheme of time-series analysis using a clustering approach to examine protein changes in sEVs and cEVs over time in SALS patients who participated in the ROPALS trial and had samples available up to 48w. **b**, **d** Plots showing log_2_(fold change) changes in each protein from each sampling time from 0w in patients in the placebo and ROPI groups (sEVs: **b**, cEVs: **d**). Clustering analysis (Ward's method, *k* = 3) of the differential changes in proteins within EVs from 0w to each sampling time in the placebo group classified proteins into three groups according to the change in each protein over time: increased (sEVs: *n* = 298, **b** left, red; cEVs: *n* = 260, **d** left, red), relatively unchanged (sEVs: *n* = 297; **b**, left, gray, cEVs: *n* = 1073, **d**, left, gray), and decreased (sEVs: *n* = 466, **b** left, blue, cEVs: *n* = 51, **d** left, blue). After clustering, changes over time in the ROPI group of proteins belonging to each clustering group were plotted (sEVs: **b** right; cEVs: **d** right). Statistical analysis was performed by two-way ANOVA followed by Bonferroni’s multiple-comparisons test. **c**, **e** GO (BP, CC, MF) and KEGG pathway analysis (cEVs: **c**, sEVs: **e**) for increased and decreased protein groups based on the log_2_(fold change) clustering results of proteins in EVs in the placebo group. *** indicates statistical significance at *P* < 0.001

Of the 20 SALS patients who participated in the ROPALS trial, patients with samples through the final week (48w) were selected (CSF: ROPI group *n* = 4, placebo group *n* = 2; serum: ROPI group *n* = 8, placebo group *n* = 3). Clustering analysis using Ward’s method was then performed for each of the sEVs and cEVs in the placebo group to determine differences in EV protein levels (log_2_(fold change)) between 0w and each sampling time point, with *k* = 3 (Fig. 4a, Supplementary Fig. S3a).

Clustering analysis of the differential change in proteins within EVs at 0w and each sampling time point in the placebo group was used to classify the change in each protein into three groups: increased (sEVs: *n* = 298, Fig. 4b, left, red, cEVs: *n* = 260, Fig. 4d, left, red), relatively unchanged (sEVs: *n* = 297, Fig. 4b, left, gray, cEVs; *n* = 1073, Fig. 4d, left, gray), and decreased (sEVs: *n* = 466, Fig. 4b, left, blue, cEVs; *n* = 51, Fig. 4d, left, blue).

Next, we examined the variation of these protein groups in the ROPI group (Fig. 4b, d, right). The increase in proteins within EVs over time in the placebo group was significantly suppressed by ROPI administration (all *P* < 0.001, Fig. 4b, d, right, red). Furthermore, these protein groups were associated with the Complement and coagulation cascades in both sEVs (KP, adj. *P* < 0.001) (Fig. 4c, left) and cEVs (KP, adj. *P* < 0.001) (Fig. 4e, left).

However, proteins for which the EV levels decreased over time in the placebo group were significantly restored by ROPI treatment (all *P* < 0.001, Fig. 4b, d, right, blue). These protein groups were involved in Platelet aggregation (KP, adj. *P* < 0.001) in sEVs (Fig. 4c, right) and antioxidant activity (MF, adj. *P* < 0.001) in cEVs (Fig. 4e right).

### Comparative analysis of DAPs, es-DAPs, and time-varying proteins

**Fig. 5:**
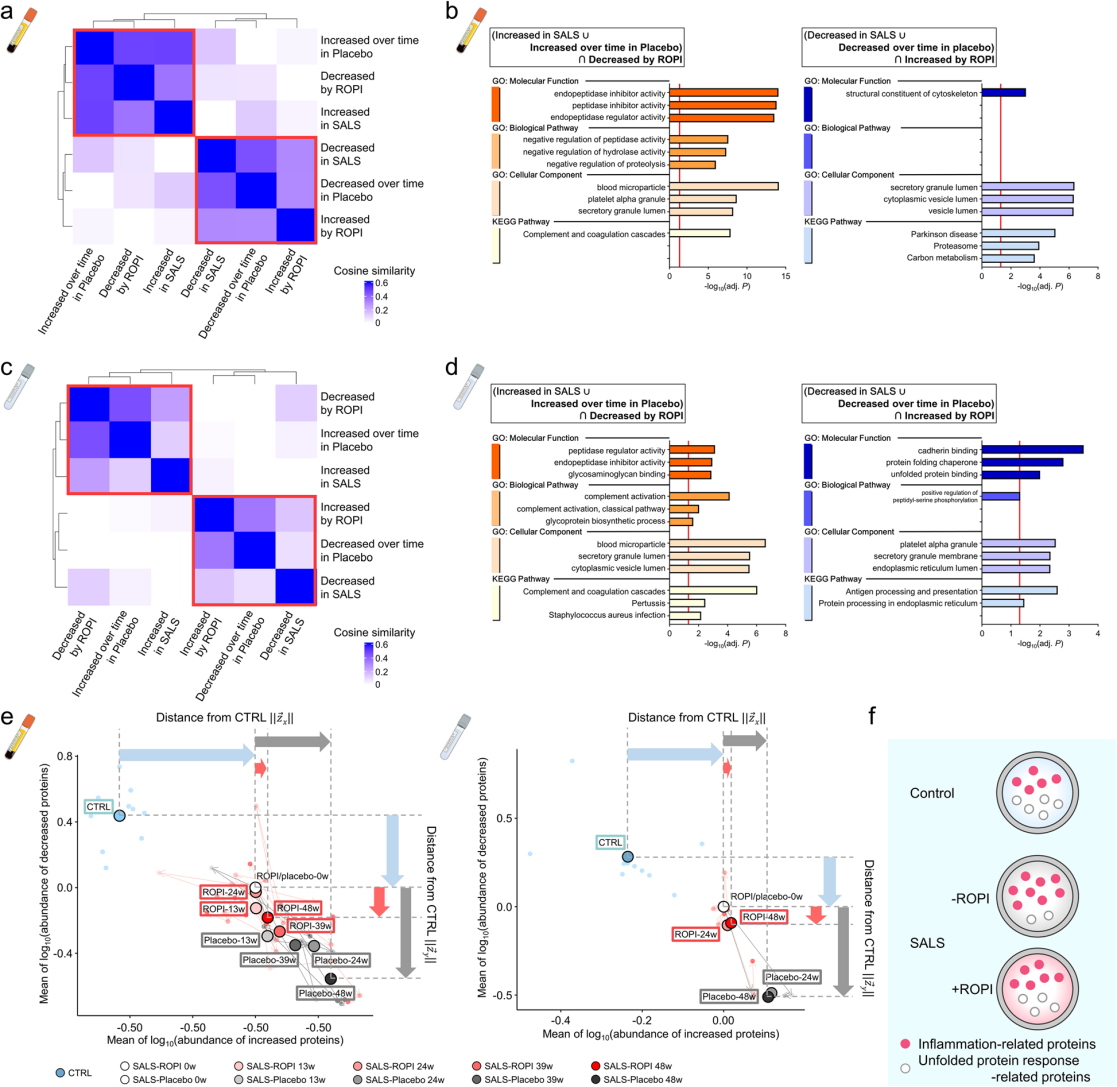
Comparison of DAPs, es-DAPs, and protein variation over time. **a**, **c** Heatmaps (sEVs: **a**, cEVs: **c**) showing cosine similarity analysis results for DAPs (control samples vs SALS patient samples), es-DAPs (placebo 0–24w vs ROPI 024w), and proteins that fluctuated over time. **b**, **d** Bar graphs showing GO (BP, CC, MF) and KEGG pathway results for proteins that increased/decreased in SALS patients or increased/decreased over time and that were decreased/increased by ROPI (sEVs: **b**, cEVs: **d**). **e** Scatter plots showing differences from 0w for protein levels that increased in SALS patients or increased over time on the vertical axis and differences from 0w for protein levels that decreased in SALS patients or decreased over time on the horizontal axis (sEVs: left, cEVs: right). Light-colored points indicate each sample, and arrows indicate trends over time. Black-bordered point show the mean for each group. The control plot is the difference between the mean protein content at 0w for each patient and the mean. **f** Schematic representation of protein composition and ROPI-induced changes in control and SALS EVs

Next, we calculated the cosine similarity of DAPs (controls vs. SALS patients), es-DAPs (placebo 0–24w vs. ROPI 0–24w), and time-varying protein groups. The increased/decreased protein groups identified in sEVs and cEVs were clearly similar, which was supported by cluster analysis (Fig. 5a, c, Supplementary Fig. S4a). Interestingly, the protein groups that increased/decreased in SALS patients or increased/decreased over time were very similar to the protein groups that decreased/increased with ROPI administration (Fig. 5a, c). These results suggest that ROPI has an anti-ALS effect on protein profiles of EVs.

The protein groups that were increased/decreased in SALS patients or over time and decreased/increased by ROPI administration were subjected to GO (MF, BP, CC) and KEGG pathway analysis. The protein group that was increased in SALS patients or increased over time and decreased by ROPI treatment was involved in the complement and coagulation cascades in both sEVs and cEVs (KP, adj. *P* < 0.001 and < 0.001, respectively). Conversely, the protein group that decreased in SALS patients or decreased over time and was increased by ROPI was enriched in Parkinson’s disease (KP, adj. *P* < 0.001) and proteasome (KP, adj. *P* = 0.0001) for sEVs (Fig. 5b) and protein folding chaperone (MF, adj. *P* = 0.0016), unfolded protein binding (MF, adj. *P* = 0.0113), and protein processing in the endoplasmic reticulum (KP, adj. *P* = 0.0344) for cEVs (Fig. 5d).

Next, we examined changes over time in SALS patients with samples through 48w in the placebo and ROPI groups to determine the mean levels of protein groups that increased/decreased in SALS patients or over time (Fig. 5e). Both the placebo and ROPI groups diverged from the control distribution over time, but the ROPI group distribution appeared to be suppressed with respect to diverging from the control distribution.

These results suggest that inflammation, represented by the complement and coagulation cascades, occurs in SALS and that the EV content of proteins involved in the UPR is decreased but that ROPI treatment ameliorates inflammation and increases the amount of UPR-related proteins (Fig. 5f).

### Reverse translational research: iPSC-derived astrocyte and iPSC-MN transcriptome changes by ROPI

**Fig. 6:**
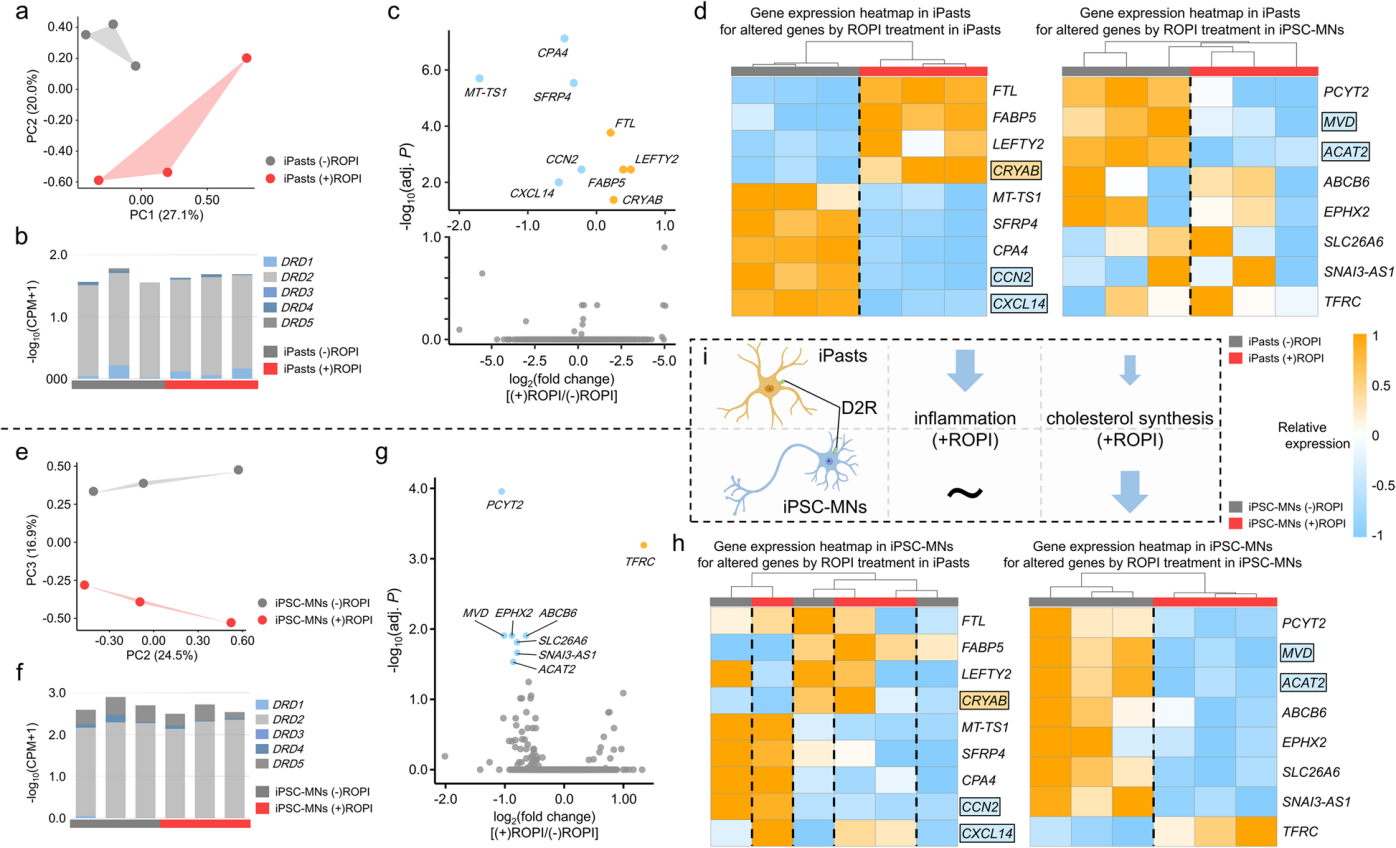
Reverse translational research results: ROPI delivery to iPasts and iPSC-MNs. **a**, **e** The results of PCA of iPast and iPSC-MN transcriptomes with and without ROPI treatment (red; ( +) ROPI, gray; ( −) ROPI). **b**, **f** Dopamine receptor expression in iPasts and iPSC-MNs with and without ROPI treatment. **c**, **g** Volcano plots showing changes in iPasts and iPSC-MNs with and without ROPI treatment. **d**, **h** Heatmaps showing the expression levels of DEGs identified in the comparative analysis of iPasts and iPSC-MNs with and without ROPI treatment. **i** Schematic representation of *DRD2* expression and ROPI-induced expression changes in iPasts and iPSC-MNs

We examined the possibility that ROPI acts on astrocytes and motor neurons to suppress neuroinflammation. First, we generated healthy human-derived iPasts and performed RNA-seq on astrocytes with or without ROPI treatment. Furthermore, we obtained RNA-seq data of healthy human-derived iPSC-MNs with and without ROPI administration from the NCBI GEO database. The PCA results showed that both the iPast and iPSC-MN transcriptomes under ROPI-treated/non-treated conditions were clearly divided into two groups, suggesting that ROPI treatment induced changes in the iPast and iPSC-MNs transcriptomes (Fig. 6a, e). Next, we confirmed sufficient expression of *DRD2* (Fig. 6b, f), suggesting that both iPasts and iPSC-MNs may be subject to D2R-dependent effects of ROPI treatment.

Additionally, we examined the transcriptome changes upon ROPI administration and identified nine and eight genes with significantly altered expression after ROPI administration in iPasts and iPSC-MNs, respectively (Fig. 6c, g). In iPasts, ROPI significantly upregulated *CRYAB* expression and downregulated expression of the chemokines *CXCL14* and *CCN2* (Fig. 6d, left). However, the expression changes induced by ROPI treatment in iPasts did not match the behavior in iPSC-MNs (Fig. 6h, left). Genes involved in cholesterol synthesis, such as *MVD* and *ACAT2*, were significantly decreased in iPSC-MNs by ROPI treatment, which showed a similar trend in iPasts (Fig. 6d, h, right). The suppressive effect of ROPI on the expression of genes involved in cholesterol synthesis was consistent with a previous report investigating iPSC-MNs from SALS patients [15].

### Biomarker search using proteins within EVs as clinical indicators of ALS

**Fig. 7:**
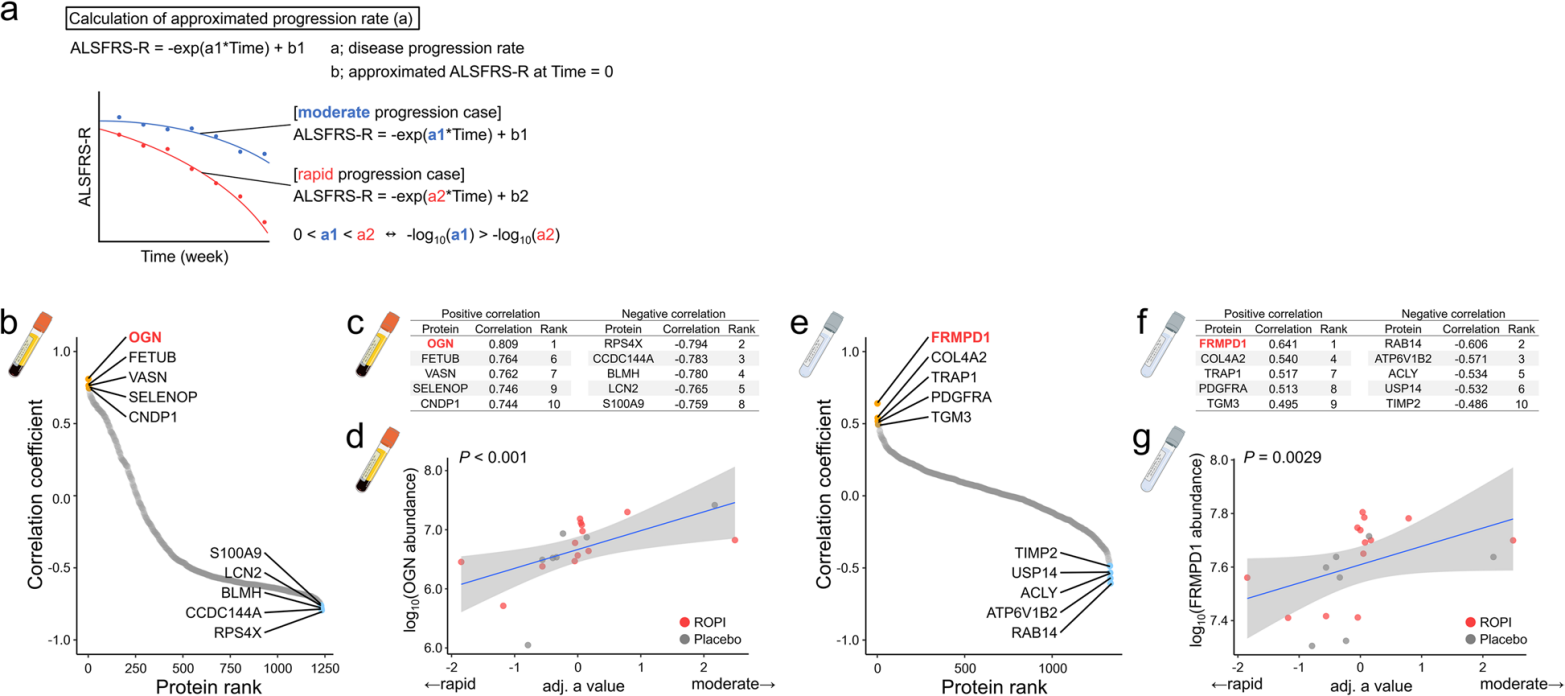
Biomarker search using proteins within EVs to identify clinical indicators of SALS. **a** Approximation equations were applied to the ALSFRS-R trends of 20 SALS patients enrolled in the ROPALS trial to calculate the aALSFRS-R. In the left figure, the middle line plot shows the measured ALSFRS-R and the predicted ALSFRS-R (aALSFRS-R) of the patients, and the right table shows the predicted results. **b**, **e** Rank plots (sEVs: **b**, cEVs: **e**) showing the correlation analysis results of the amount of each protein in sEVs or cEVs at 0w and the subsequent rate of adaptation progression (normalized *a* value). Colors indicate the top five positive and negative proteins. **c and f** Tables of the top five positive and negative proteins (cEVs: **c**, sEVs: **f**). **d**, **g** Scatter plots showing the proteins with the highest prediction accuracy compared with the different indices (cEVs: **d**, sEVs: **g**)

Based on the detailed clinical patient data collected in the ROPALS trial, we explored biomarkers for clinical indication of ALS using proteins within EVs. First, we approximated the change in the ALSFRS-R, a key clinical indicator of ALS symptoms, during the ROPALS trial with the model equation $ALSFRS$-$R=\text{exp}\left(a*week\right)+b$. z-Score transformed $a$ values (adj. $a$ values) within each ROPI and placebo group were calculated using $-{\text{log}}_{10}a$, where $a$ represents the disease progression rate for each patient (Fig. 7a). Higher adj. $a$ values indicate slower disease progression. The aALSFRS-R was calculated from the coefficients $a$ and $b$ at each sampling time. The fixed-point disease progression rate was calculated by differentiating the model equation at each sampling time.

Correlation analysis was performed between the content of each protein in EVs at 0w and the adj. $a$ value (sEVs: Fig. 7b, cEVs: Fig. 7e). Osteoglycin (OGN) and FERM and PDZ domain containing 1 (FRMPD1) were identified as the markers that best predicted the adj. $a$ values, i.e., the prognostic values of sEVs (*R* = 0.809, *P* < 0.001, Fig. 7c, d) and cEVs (*R* = 0.641, *P* = 0.0029, Fig. 7f, g).

Furthermore, correlation analyses were performed between each protein in EVs at each sampling time, the aALSFRS-R (Supplementary Figure S5a–f), and the progression rate of the aALSFRS-R at that time (Supplementary Fig. S5g–l).

### Diagnostic biomarker search using machine learning and machine learning-based classifier generation

Machine learning methods were used to build classifiers to discriminate between samples from controls and SALS patients based on the EV protein composition.

**Fig. 8:**
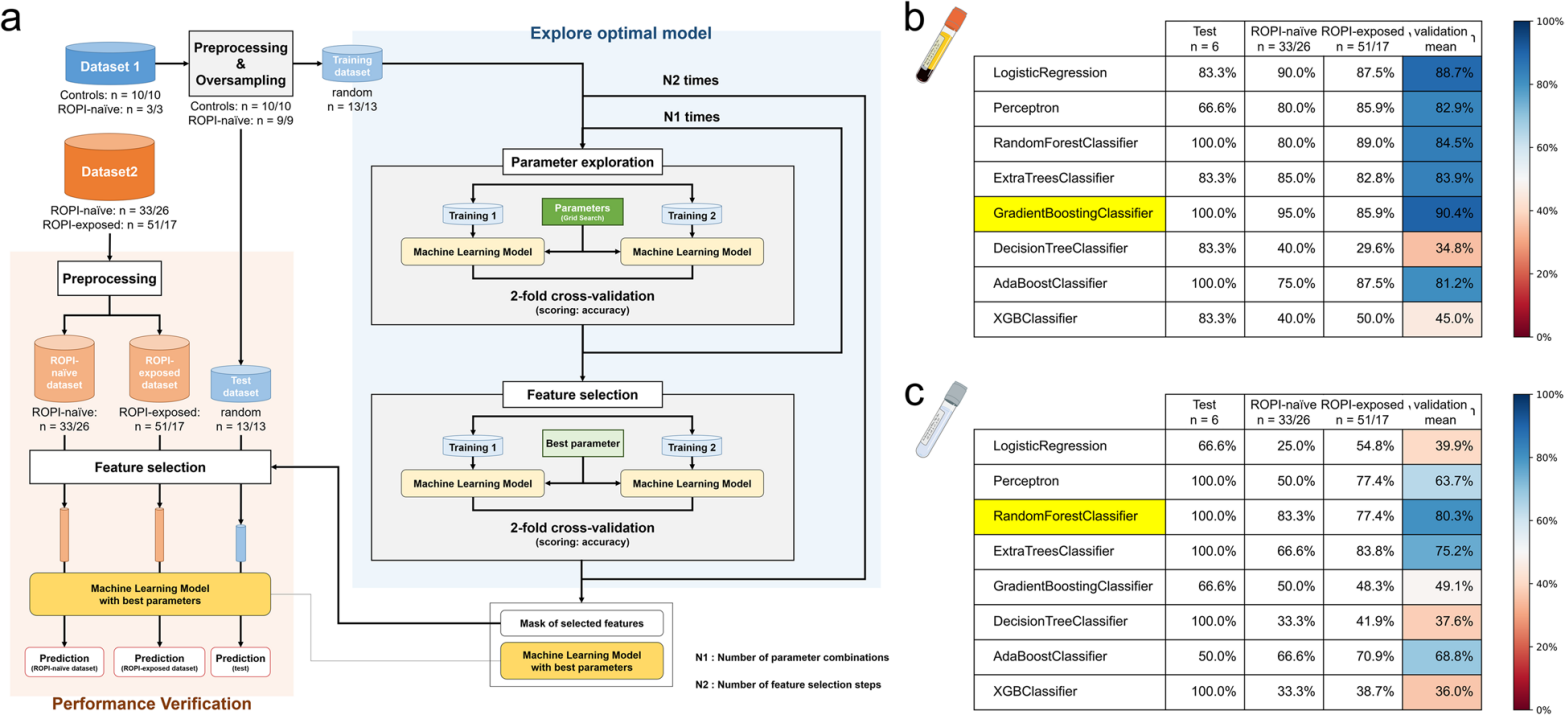
Exploration of diagnostic biomarkers using machine learning methods and generation of machine learning-based classifiers. **a** Schematic of the learning framework for a limited sample size and unbalanced datasets. This framework consists of three steps: oversampling, parameter searches, and feature selection. **b**, **c** Tables and heatmaps showing the accuracy of the trained models according to protein composition (sEVs: **b** and cEVs: **c**). The test dataset contains an equal number of samples from controls and SALS patients (ROPI-naive). The ROPI-naive dataset consists of samples collected from SALS patients before they received ROPI, and the ROPI-exposed dataset consists of samples taken from SALS patients after they received ROPI. Accuracy is shown by %, and the average accuracy for the ROPI-naive and ROPI-exposed datasets is shown in the heatmap

The accuracy of each model on the test sEV dataset was 100% for the Random Forest Classifier, Gradient Boosting Classifier, and AdaBoost Classifier. Furthermore, the Gradient Boosting Classifier had the highest average accuracy for each model for validation dataset 1 (ROPI-naive) and validation dataset 2 (ROPI-exposed), which contain different batches from the training and test datasets, with 155 proteins selected as features and an accuracy of 90.4% (Fig. 8b).

The accuracy of each model for the cEV test dataset was 100% for Perceptron and the Random Forest, Extra Trees, Decision Tree, and XBG Classifiers. Furthermore, the Random Forest Classifier had the highest average accuracy for each model for the validation datasets, with 19 proteins selected as features and an accuracy of 80.3% (Fig. 8c).

The lists of features selected by each model are provided in Supplementary Table S4.

## Discussion

A key finding of our study is the characterization of the protein profiles of sEVs and cEVs from SALS patients, marked by upregulation of inflammation-related proteins, such as those involved in the complement and coagulation cascades, and downregulation of proteins associated with the UPR, compared with those in controls. This alteration manifested in a pathological progression-dependent manner over time (Fig. 2a–e). Specifically, detection of SALS-specific proteins such as immunoglobulin complexes and C-reactive protein in cEVs from SALS patients (Supplementary Fig. S1b, Supplementary Table S2) indicates inflammation within the CSF of these patients. These proteins are known activators of complement pathways, particularly the classical pathway, a finding consistent with the present results. Past research demonstrated similar increases in inflammatory complement proteins in astrocyte-derived EVs from Alzheimer’s disease patients [48, 49] and animal models of Gulf War illness [50]. These findings suggest that neurodegeneration-linked neuroinflammation augments complement-related proteins in EVs, which might exacerbate neurological damage [51].

In contrast, sEVs and cEVs from SALS patients have diminished levels of proteins related to the UPR (Fig. 2e). Prior research aligns with our findings, indicating a decrease in sEV HSP90 levels in ALS patients (without distinguishing between familial ALS and SALS) [52], which is consistent with our results. In vitro studies suggest UPR-related proteins, including the HSP family, mitigate TDP-43 aggregation [53–55]. Of note, UPR-related proteins, represented by the HSP70 and HSP90 families, which are transported by EVs, enhance the protein folding environment in recipient cells [56]. Astrocytes also release HSP70-rich EVs under stress conditions [57]. These observations suggest EVs delivering HSP and other UPR proteins exert a neuroprotective effect. The decline in UPR-related proteins in EVs from SALS patients could indirectly promote TDP-43 aggregation in motor neurons, worsening SALS pathology. Interestingly, UPR-related protein levels were reduced in both sEVs and cEVs, suggesting that decreases in these proteins occur not only in the brain and spinal cord, but also in circulating EVs throughout the body, may be involved in the pathogenesis of SALS.

On the other hand, the increase in complement- and coagulation-related proteins and the decrease in UPR-related proteins in EVs observed in this study may be common to some extent in neurodegenerative diseases. As mentioned earlier, the increase in inflammation-related proteins in EVs is associated with neuroinflammation. In neurodegenerative diseases, inflammatory responses are triggered by neurodegeneration [58], so the increase in complement- and coagulation-related proteins in EVs may not be specific to SALS, but may be a phenomenon common to some extent in neurodegenerative diseases. Similarly, a decrease in UPR-associated proteins may contribute to the disruption of proteostasis in neurons. The possibility cannot be ruled out that decreased UPR-associated proteins may be a common phenomenon in neurodegenerative diseases, where the accumulation of denatured proteins is a common phenomenon. Future comprehensive studies of protein composition in EVs of not only SALS but also other neurodegenerative diseases, such as Alzheimer’s disease and Parkinson’s disease, may advance our understanding of neurodegenerative diseases as a whole.

Further comparisons of sEV and cEV protein compositions revealed a similar disease-related trend in protein group variations (Fig. 1f). The sEV and cEV protein content showed striking similarity in SALS patients and controls, at least partly, suggestive of elevated blood–brain barrier permeability in SALS patients. This observation aligns with previous reports of increased blood–brain barrier permeability in ALS patients [59–61], suggesting that peripheral blood-derived EVs might be useful for diagnosing and treating central nervous system lesions [62].

Another significant finding of the present study is that ROPI administration modulated pathological changes in EV protein profiles toward control or early disease states. ROPI, a D2R agonist, may be responsible for the observed decrease in inflammation-related protein groups. Multiple studies support crucial roles of D2R in attenuating neuroinflammation by microglia and astrocytes [63–65]. Activation of D2R is thought to suppress these nuclear translocations by inducing increased expression of *CRYAB* and promoting binding of CRYAB to STAT3 and NF-κB in the cytoplasm (Supplementary Fig. 6) [66, 67].

In the present study, sufficient DRD2 expression in iPasts was confirmed in healthy controls (Fig. 6b), and ROPI treatment significantly increased *CRYAB* expression and decreased *CCN2* and *CXCL14* expressions (Fig. 6c, d). As mentioned earlier, in astrocytes, D2R stimulation regulates innate immunity through CRYAB, which suppresses neuroinflammation [63, 65]. Conversely, CCN2, as the CTGF/CCN2 complex, increases the expression of inflammatory genes including chemokines and cytokines and enhances inflammatory responses through astrocyte activation [68, 69]. Additionally, CTGF was significantly upregulated in reactive astrocytes in the anterior horn and white matter of the spinal cord in SALS and familial ALS patients [70]. The neuroinflammatory role of CXCL14 is not well understood, but its overexpression exacerbates collagen-induced arthritis, and its addition to dendritic cells results in increased NF-κB activity and their activation [71, 72], suggesting its involvement in inflammatory responses. However, the ROPI-induced expression changes in iPSC-MNs were not clearly consistent with those in iPasts (Fig. 6c, d, g, h), suggesting that the reduction of inflammation-related proteins in EVs by ROPI administration may result from its action on cells other than motor neurons, such as astrocytes. These results and previous studies strongly support the possibility that ROPI suppresses neuroinflammation via D2R stimulation and suppresses the complement and coagulation-related protein levels in EVs. Furthermore, reducing inflammation and decreasing the levels of proteins involved in the complement and coagulation cascades in ALS patients may be a potential therapeutic target for ALS.

We also examined biomarkers for various clinical indicators of ALS. Among all candidates in sEVs and cEVs, OGN (also known as mimecan) in sEVs was the best marker for predicting disease progression. OGN is a tandem leucine-rich repeat-containing protein that plays a critical role in extracellular matrix assembly [73, 74] and affects bone formation and fibrogenesis [75, 76]. Interestingly, OGN significantly increased IGF-2/IGFB2-induced neurite outgrowth [77]. In this study, sEVs with higher OGN levels at baseline were associated with slower disease progression and a better prognosis, raising the possibility that OGN in EVs in peripheral blood may have anti-ALS effects on neurons. However, the possibility that these results are simply correlations cannot be excluded given the small sample size of this study and that a comprehensive protein search in EVs was conducted. Furthermore, it remains unclear whether these candidate biomarker proteins are transported into MNs by EVs from other cell types, and if so, whether they have functional roles within MNs. To investigate this, one approach would be to generate non-MN central nervous system cells, such as astrocytes, oligodendrocytes, and microglia, from iPSCs and co-culture them with MNs [78, 79]. This method would facilitate the study of the influence of non-MN cells in ALS, with a particular focus on EV-mediated protein trafficking. Therefore, further large-scale studies are needed to determine whether these biomarkers are useful and of biological significance.

Additionally, this study evaluated the performance of trained machine learning diagnostic models on the protein profiles of sEV and cEV datasets. The Gradient Boosting Classifier performed well on the sEV dataset (selected features, 155; accuracy on the test dataset, 100%; accuracy on the validation dataset, 90.4%), and the Random Forest Classifier performed best on the cEV dataset (selected features, 19; accuracy on the test dataset, 100%; accuracy on the validation dataset, 80.3%). Because the test dataset contained approximately half of the control samples, we estimate that these trained models can discriminate between control and SALS patient samples with high accuracy. Furthermore, SALS data measured in different batches could also be discriminated with high accuracy, suggesting the versatility of these trained models. Interestingly, classification by the machine learning model was more accurate for sEVs than for cEVs, suggesting that blood, which can be collected less invasively, can be used with sufficient accuracy to diagnose SALS. However, several factors need to be considered in this study. First, the sample size is small, which may affect the robustness of the results. Second, batch-to-batch differences may affect the accuracy of the results. Third, since this study was based on a clinical trial consisting of 60 weeks, it was not possible to include longer-term prognosis in the analysis. Finally, the selected features do not always guarantee biological interpretability. Therefore, further validation by larger and longer-term studies is essential to develop diagnostics based on protein composition in EVs and their clinical application.

Most previous ALS studies related to proteins in fluid-derived EVs have focused on TDP-43 in EVs. Detection of TDP-43 in EVs is important for the diagnosis and understanding of the pathophysiology of ALS because EVs act as “garbage cans” that release intracellularly accumulated abnormal proteins. However, by identifying a comprehensive protein composition in EVs, this study revealed that various functional proteins are altered in SALS, strongly suggesting that these proteins may contribute to disease pathogenesis. Furthermore, the protein composition changed significantly with disease progression over time, which was suppressed by ROPI administration.

## Conclusions

In conclusion, our comprehensive EV protein composition analysis revealed various functional protein alterations in SALS, potentially contributing to its pathogenesis. Furthermore, this composition changed significantly over time, which could be mitigated by ROPI administration. Our approach may prove invaluable for understanding ALS pathogenesis and drug mechanisms and awaits validation in future large-scale studies.

### Supplementary Information

Supplementary Material 1.

Supplementary Material 2.

Supplementary Material 3.

Supplementary Material 4.

Supplementary Material 5. Supplementary Figure S1. Identification and analysis of SALS-specific proteins in EVs. a and b Scatterplot showing detection rates of proteins identified in sEVs and cEVs in controls and ROPI-naive SALS patient samples (sEVs: a, cEVs: b). Proteins with detection rates greater than 90% in EVs derived from SALS patients and less than 10% in EVs derived from controls are defined as SALS-specific proteins. Bar graphs show GO term (BP, CC, MF) and KEGG pathway analysis results for SALS-specific proteins.

Supplementary Material 6. Supplementary Figure S2. Comparative analysis of protein profiles within EVs from controls and SALS patients. a and b The clustering analysis results of each term in GO (BP) and KEGG pathway analyses (sEVs: a, cEVs: b) for DAPs obtained by comparing the log_2_(fold change) of placebo 0–24w and ROPI 0–24w samples. Each node represents a term, the color of each node represents the q value of each term, and blue edges represent gene overlap.

Supplementary Material 7. Supplementary Figure S3. Clustering analysis for changes in proteins within EVs over time in the placebo group. a Clustering analysis of proteins within EVs in the placebo group was performed using Ward's method with *k* = 3 for changes over time according to the log_2_(fold change) between 0w and at each sampling time. Dendrograms show the clustering analysis results in sEVs and sEVs.

Supplementary Material 8. Supplementary Figure S4. Cosine similarity analysis results of sEVs and cEVs. a Heat map showing the cosine similarity analysis results for DAPs (control samples vs SALS patient samples), es-DAPs (placebo 0–24w vs ROPI 0–24w), and proteins that increased/decreased over time in sEVs and cEVs.

Supplementary Material 9. Supplementary Figure S5. Biomarker search for clinical indicators of ALS using proteins within EVs. a-l Rank plots (sEVs: a and g, cEVs: d and j) showing the correlation analysis results of the amount of each protein contained in sEVs or cEVs at each time point and the aALSFRS-R (a-f) and the progression speed at a fixed point (g–l). A cut-off value of 0.5 was set for the correlation coefficient, and the table shows the top five proteins (sEVs: b and h, cEVs: e and k). Scatter plots (sEVs: c and i, cEVs: f and l) show the proteins with the highest prediction accuracy compared with the different indices.

Supplementary Material 10. Supplementary Figure S6. Schematic illustration showing the molecular mechanism of neuroinflammation suppression by the D2R-CRYAB pathway. a Activation of D2R is thought to suppress these nuclear translocations by inducing increased expression of CRYAB and promoting binding of CRYAB to STAT3 and NF-κB in the cytoplasm.

## Data Availability

All raw data and codes are available from the corresponding author upon reasonable request. The RNA-seq data were deposited in the NCBI GEO database (GEO accession number GSE242978).

## Abbreviations

aALSFRS-R: Approximated revised amyotrophic lateral sclerosis functional rating scale
adj. *P*: Adjusted *P*
ALS: Amyotrophic lateral sclerosis
ALSFRS-R: Revised amyotrophic lateral sclerosis functional rating scale
BP: Biological pathway
CC: Cellular component
cEVs: Cerebrospinal fluid-derived extracellular vesicles
CSF: Cerebrospinal fluid
CTRL: Control
D2R: Dopamine D2 receptor
DAPs: Differentially abundant proteins
DEGs: Differentially expressed genes
DR: Detected rate
es-DAPs: Effect size-based differentially abundant proteins
EVs: Extracellular vesicles
FRMPD1: FERM and PDZ domain containing 1
GEO: Gene Expression Omnibus
GO: Gene Ontology
iPast: Induced pluripotent stem cell-derived astrocyte
iPSC: Induced pluripotent stem cell
iPSC-MNs: Induced pluripotent stem cell-derived spinal motor neurons
KEGG: Kyoto Encyclopedia of Genes and Genomes
LC/MS: Liquid chromatography/mass spectrometer
MF: Molecular function
NCNP: National Center of Neurology and Psychiatry (Japan)
OGN: Osteoglycin
PCA: Principal component analysis
RNA-seq: Ribonucleic acid sequencing
ROPALS trial: Ropinirole Hydrochloride Remedy for Amyotrophic Lateral Sclerosis trial
ROPI: Rpinirole hydrochloride
s.d.: Standard deviation
SALS: Sporadic amyotrophic lateral sclerosis
sEVs: Serum-derived extracellular vesicles
UMAP: Uniform manifold approximation and projection
UPR: Unfolded protein response
Valid: Validation dataset
w: Week
