# Supplementary figures and images for: Proteomic insights into extracellular vesicles in ALS for therapeutic potential of Ropinirole and biomarker discovery
Source: Inflamm Regen. 2024 Jul 12;44:32. doi: 10.1186/s41232-024-00346-1 (PMC11241965; doi:10.1186/s41232-024-00346-1)

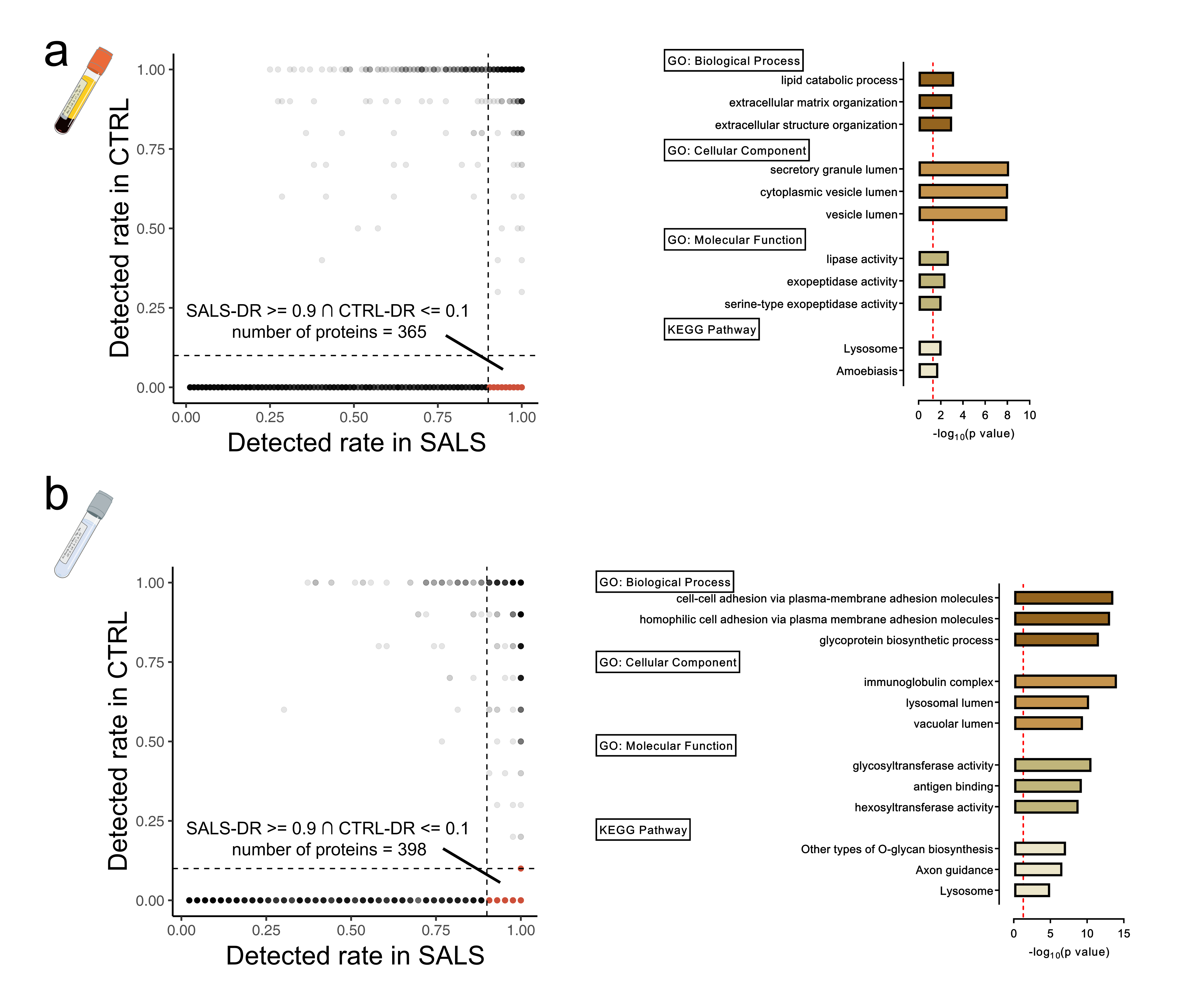

Supplement: Supplementary file 5 — Supplementary Material 5. Supplementary Figure S1. Identification and analysis of SALS-specific proteins in EVs. a and b Scatterplot showing detection rates of proteins identified in sEVs and cEVs in controls and ROPI-naive SALS patient samples (sEVs: a, cEVs: b). Proteins with detection rates greater than 90% in EVs derived from SALS patients and less than 10% in EVs derived from controls are defined as SALS-specific proteins. Bar graphs show GO term (BP, CC, MF) and KEGG pathway analysis results for SALS-specific proteins. [file 41232_2024_346_MOESM5_ESM.tif]

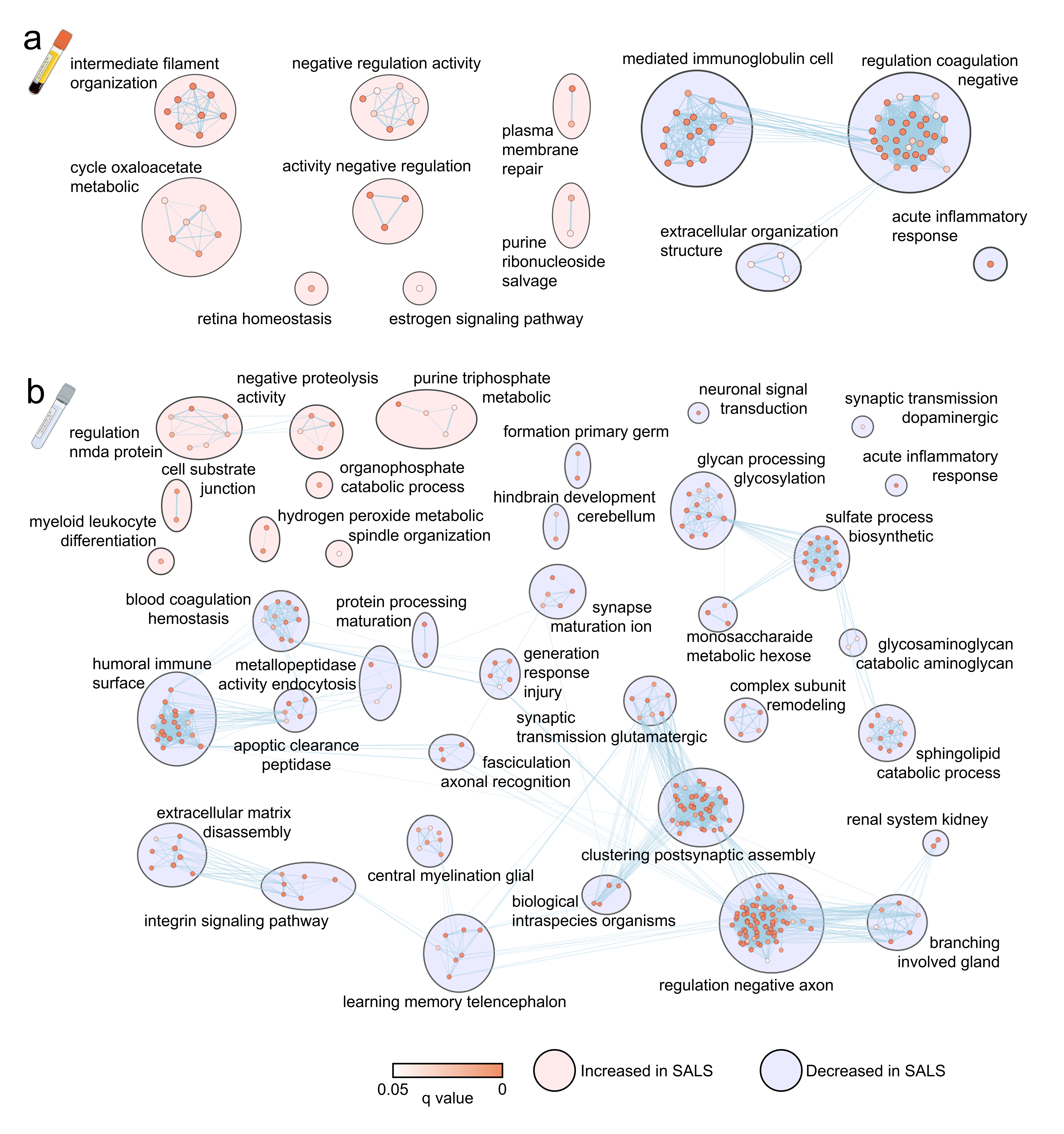

Supplement: Supplementary file 6 — Supplementary Material 6. Supplementary Figure S2. Comparative analysis of protein profiles within EVs from controls and SALS patients. a and b The clustering analysis results of each term in GO (BP) and KEGG pathway analyses (sEVs: a, cEVs: b) for DAPs obtained by comparing the log2(fold change) of placebo 0–24w and ROPI 0–24w samples. Each node represents a term, the color of each node represents the q value of each term, and blue edges represent gene overlap. [file 41232_2024_346_MOESM6_ESM.tif]

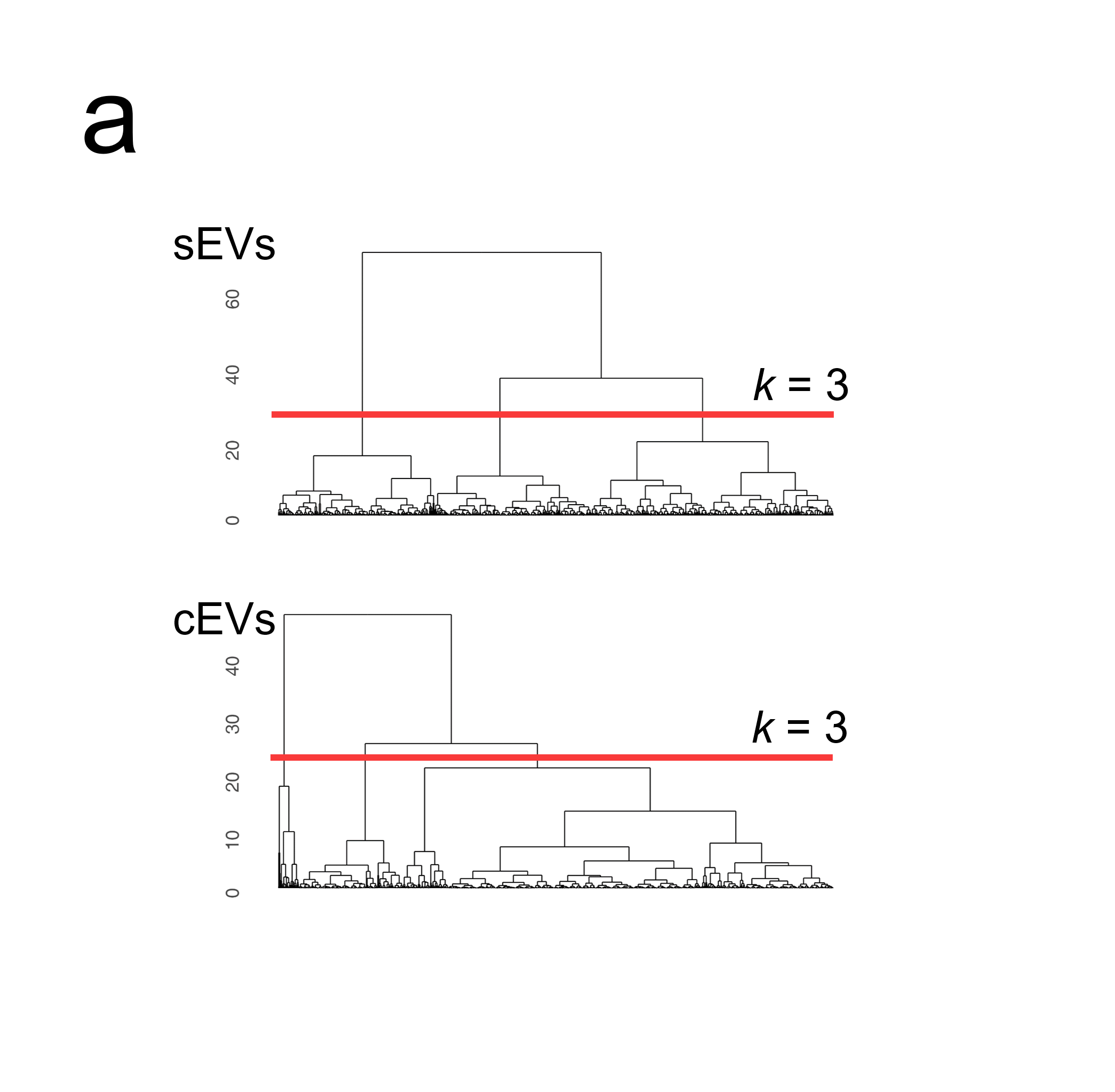

Supplement: Supplementary file 7 — Supplementary Material 7. Supplementary Figure S3. Clustering analysis for changes in proteins within EVs over time in the placebo group. a Clustering analysis of proteins within EVs in the placebo group was performed using Ward's method with k = 3 for changes over time according to the log2(fold change) between 0w and at each sampling time. Dendrograms show the clustering analysis results in sEVs and sEVs. [file 41232_2024_346_MOESM7_ESM.tif]

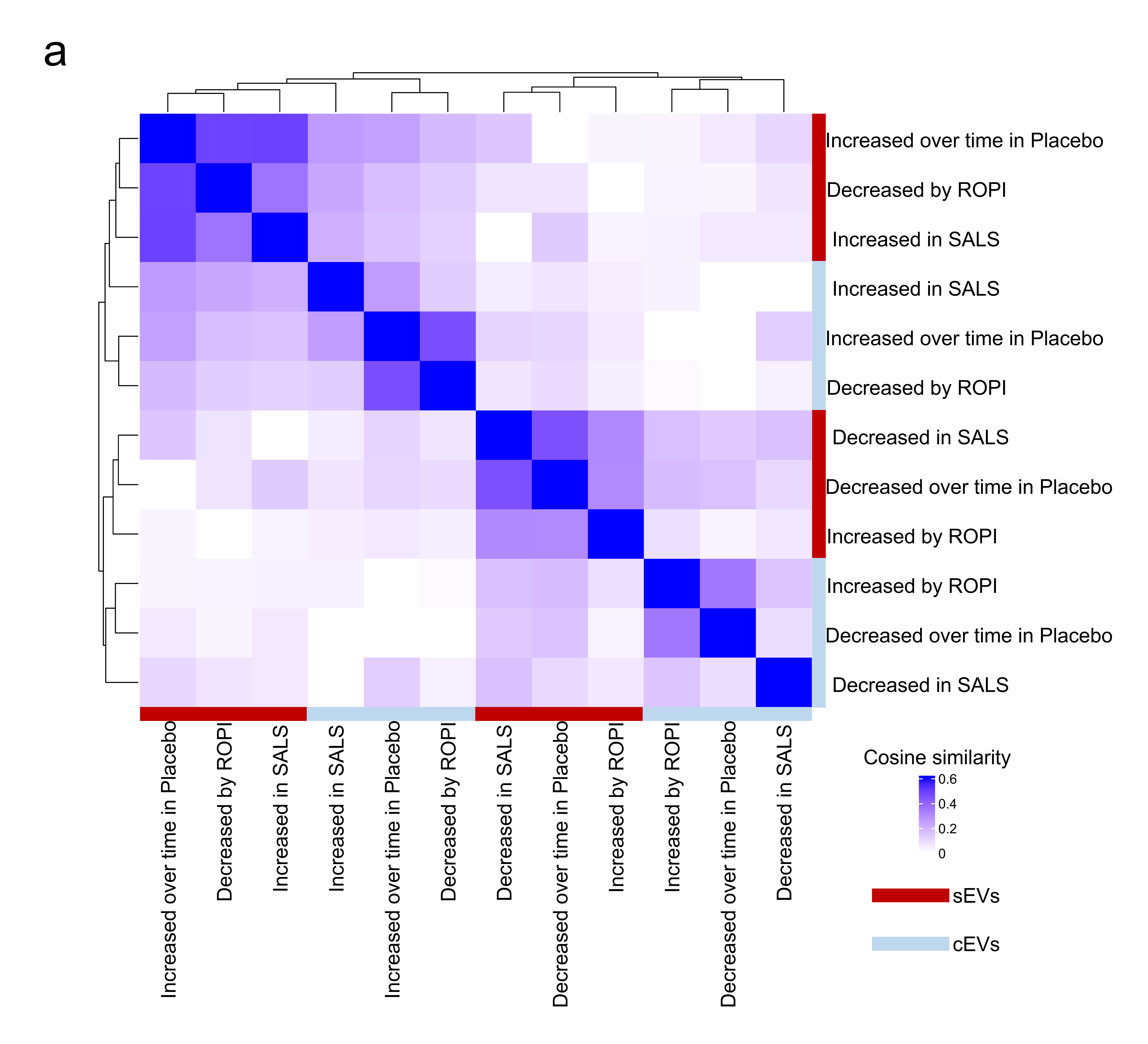

Supplement: Supplementary file 8 — Supplementary Material 8. Supplementary Figure S4. Cosine similarity analysis results of sEVs and cEVs. a Heat map showing the cosine similarity analysis results for DAPs (control samples vs SALS patient samples), es-DAPs (placebo 0–24w vs ROPI 0–24w), and proteins that increased/decreased over time in sEVs and cEVs. [file 41232_2024_346_MOESM8_ESM.tif]

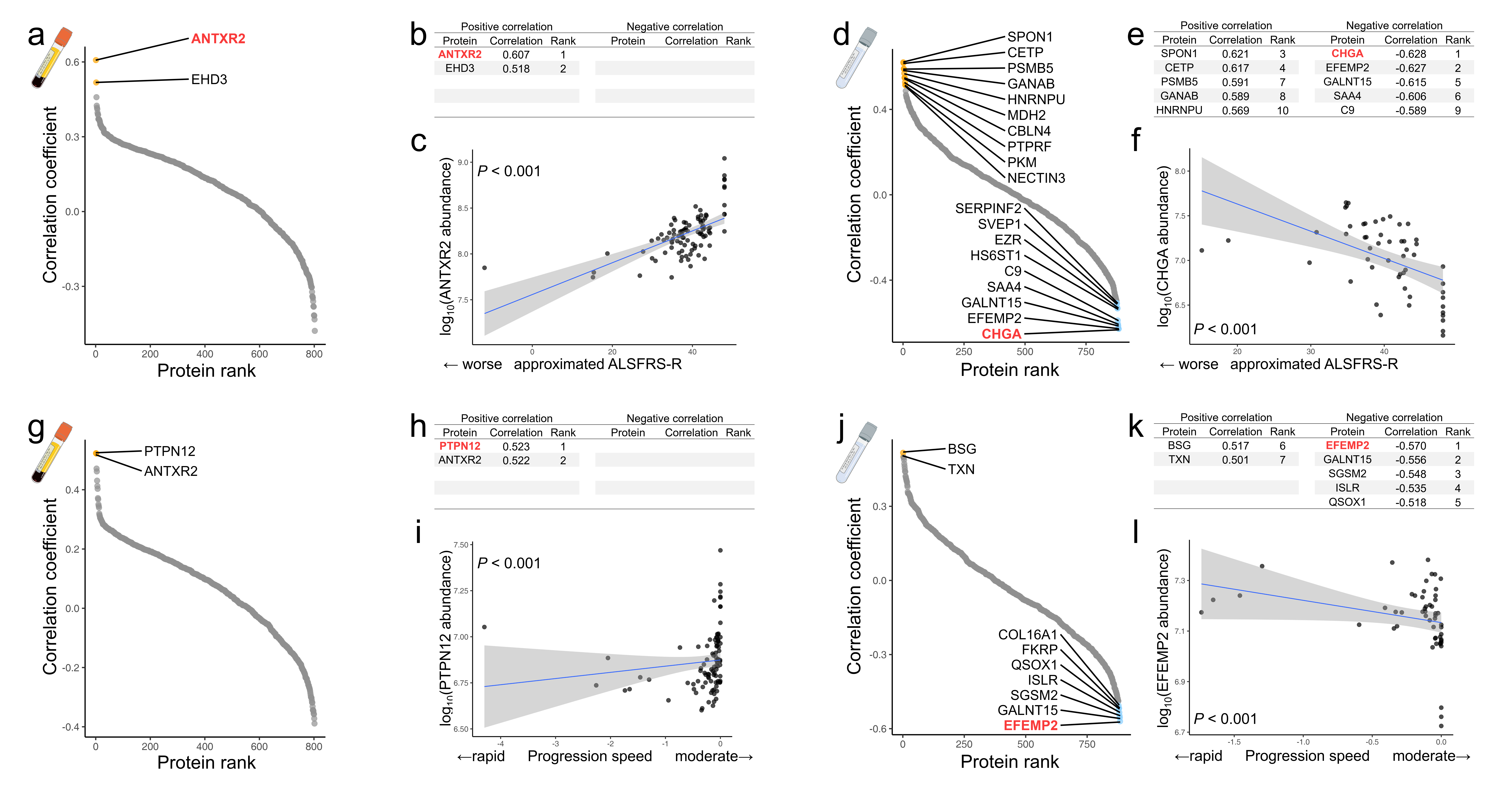

Supplement: Supplementary file 9 — Supplementary Material 9. Supplementary Figure S5. Biomarker search for clinical indicators of ALS using proteins within EVs. a-l Rank plots (sEVs: a and g, cEVs: d and j) showing the correlation analysis results of the amount of each protein contained in sEVs or cEVs at each time point and the aALSFRS-R (a-f) and the progression speed at a fixed point (g–l). A cut-off value of 0.5 was set for the correlation coefficient, and the table shows the top five proteins (sEVs: b and h, cEVs: e and k). Scatter plots (sEVs: c and i, cEVs: f and l) show the proteins with the highest prediction accuracy compared with the different indices. [file 41232_2024_346_MOESM9_ESM.tif]

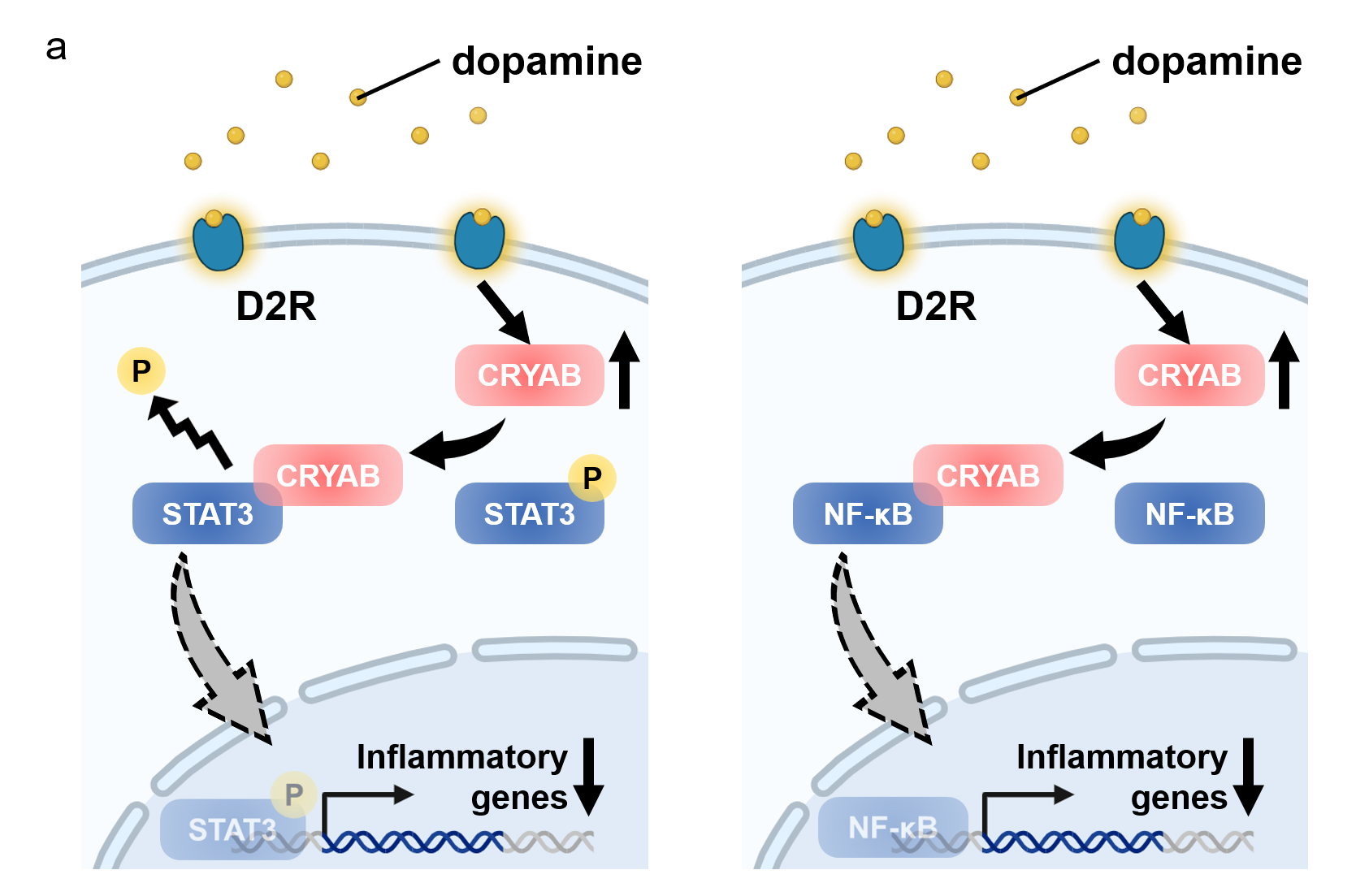

Supplement: Supplementary file 10 — Supplementary Material 10. Supplementary Figure S6. Schematic illustration showing the molecular mechanism of neuroinflammation suppression by the D2R-CRYAB pathway. a Activation of D2R is thought to suppress these nuclear translocations by inducing increased expression of CRYAB and promoting binding of CRYAB to STAT3 and NF-κB in the cytoplasm. [file 41232_2024_346_MOESM10_ESM.tif]
